# Inferring the effective TOR-dependent network: a computational study in yeast

**DOI:** 10.1186/1752-0509-7-84

**Published:** 2013-08-30

**Authors:** Shahin Mohammadi, Shankar Subramaniam, Ananth Grama

**Affiliations:** 1 Department of Computer Science, Purdue University, West Lafayette, Indiana, USA; 2 Department of Bioengineering, University of California at San Diego, La Jolla, California, USA

**Keywords:** Target of rapamycin (TOR), Yeast aging, Interactome, Information flow analysis, Effective response network

## Abstract

**Background:**

Calorie restriction (CR) is one of the most conserved non-genetic interventions that extends healthspan in evolutionarily distant species, ranging from yeast to mammals. The target of rapamycin (TOR) has been shown to play a key role in mediating healthspan extension in response to CR by integrating different signals that monitor nutrient-availability and orchestrating various components of cellular machinery in response. Both genetic and pharmacological interventions that inhibit the TOR pathway exhibit a similar phenotype, which is not further amplified by CR.

**Results:**

In this paper, we present the first comprehensive, computationally derived map of TOR downstream effectors, with the objective of discovering key lifespan mediators, their crosstalk, and high-level organization. We adopt a systematic approach for tracing information flow from the TOR complex and use it to identify relevant signaling elements. By constructing a high-level functional map of TOR downstream effectors, we show that our approach is not only capable of recapturing previously known pathways, but also suggests potential targets for future studies.

Information flow scores provide an aggregate ranking of relevance of proteins with respect to the TOR signaling pathway. These rankings must be normalized for degree bias, appropriately interpreted, and mapped to associated roles in pathways. We propose a novel statistical framework for integrating information flow scores, the set of differentially expressed genes in response to rapamycin treatment, and the transcriptional regulatory network. We use this framework to identify the most relevant transcription factors in mediating the observed transcriptional response, and to construct the *effective response network* of the TOR pathway. This network is hypothesized to mediate life-span extension in response to TOR inhibition.

**Conclusions:**

Our approach, unlike experimental methods, is not limited to specific aspects of cellular response. Rather, it predicts transcriptional changes and post-translational modifications in response to TOR inhibition. The constructed effective response network greatly enhances understanding of the mechanisms underlying the aging process and helps in identifying new targets for further investigation of anti-aging regimes. It also allows us to identify potential network biomarkers for diagnosis and prognosis of age-related pathologies.

## Background

Cellular aging is a multi-factorial complex phenotype, characterized by the accumulation of damaged cellular components over the organism’s life-span [1]. The progression of aging depends on both the increasing rate of damage to DNA, RNA, proteins, and cellular organelles, as well as the gradual decline of cellular defense mechanisms against stress. This can ultimately lead to a dysfunctional cell with a higher risk factor for disease.

Limiting caloric intake without causing malnutrition, also known as *calorie restriction (CR)*, is one of the most conserved non-genetic interventions, which extends life-span in evolutionarily distant species ranging from yeast to mammals [1-3]. Inhibition of the nutrient-sensing pathways, using either genetic or pharmacological intervention, also results in a similar phenotype [1,2]. More importantly, increased lifespan is accompanied by an increased *healthspan*, which delays both the progression and the increasing risk-factor for a wide range of age-related diseases, including cancers [4-7], cardiovascular disease [8-11], and multiple neurodegenerative disorders [12-17]. The extent to which these pathologies share their underlying biology is a topic of active investigation. Emerging evidence, however, supports the hypothesis that large classes of age-related diseases are driven by similar underlying mechanisms [18]. Understanding and controlling these mechanisms, therefore, constitute critical aspects of preventing or delaying the onset of age-related pathologies. Motivated by these observations, considerable effort has been invested in understanding the downstream effectors of the nutrient-sensing pathways that orchestrate CR-mediated life-span extension.

The budding yeast, *Saccharomyces cerevisiae*, has been used extensively as a model organism in aging research, due to its rapid growth and ease of manipulation [3,19]. Having two different aging paradigms – replicative life-span (RLS), defined as “the number of buds a mother cell can produce before senescence occurs”, and chronological life-span (CLS), defined as “the duration of viability after entering the stationary-phase”, yeast provides a unique opportunity for modeling both proliferating and post-mitotic cells. Understanding the underlying mechanisms driving RLS and CLS can ultimately be used to shed light on the progression of cancers and neurodegenerative diseases, respectively.

Yeast cells are typically cultured in growth media containing 2% glucose. Reducing glucose concentration to 0.5% or less is one of the best characterized CR regimens in yeast, which increases both CLS and RLS [20-22]. The target of rapamycin (TOR) has been shown to play a key role in mediating the observed life-span extension in response to CR [23]. TOR is a serine/threonine protein kinase, which belongs to the conserved family of PI3K-related kinases (PIKKs). It was first identified using genetic studies in yeast while searching for mutants that confer rapamycin-resistance [24]. It was later discovered that TOR protein kinases, encoded by TOR1 and TOR2 genes in yeast, form two structurally and functionally distinct multiprotein complexes [25-28]. TOR Complex 1 (TORC1) is rapamycin-sensitive and consists of both TOR proteins, TOR1 and TOR2, together with KOG1, LST8, and TCO89. On the other hand, TOR Complex 2 (TORC2) does not contain TOR1, is not inhibited by rapamycin, and contains AVO1, AVO2, AVO3, LST8, BIT2, and BIT61. These two complexes correspond to two separate branches of the TOR signaling network, controlling the spatial and temporal aspects of cell growth, respectively, which are conserved from yeast to humans [28]. Interestingly, TORC1 also has a critical role in aging and age-related pathologies [29,30]. Many of the known oncoproteins act as upstream activators of TORC1, while several tumor suppressor proteins inhibit its activity [31,32]. From a systems point of view, TORC1 acts as a hub that integrates various nutrient and stress-related signals and regulates a variety of cellular responses [33-35]. Inhibiting TOR signaling using rapamycin provides a unique opportunity to identify its downstream effectors. However, these targets may be regulated in different ways, including, but not limited to, transcription regulation, translational control, and post-translational modifications. Capturing various changes that happen during rapamycin treatment, in order to create a comprehensive systems view of the cellular response, is a complex task.

In this paper, we propose a complementary, computational approach to reconstruct a comprehensive map of TOR downstream effectors. We develop a systematic approach to couple random walk techniques with rigorous statistical models, integrate different datasets, and identify key targets in calorie restriction that are mediated by TOR pathway. Using GO enrichment analysis of high scoring nodes, we show that information flow analysis not only identifies previously known targets of TORC1, but also predicts new functional roles for further studies. We cross-validate our results with transcriptome profile of yeast in response to rapamycin treatment and show that our method can accurately predict transcriptional changes in response to TORC1 inhibition. Information flow scores provide an aggregate ranking of proteins, with respect to their relevance to the TOR signaling pathway, and are highly susceptible to degree bias. To remedy this and to elucidate the roles of underlying signaling elements, we propose a novel statistical framework for integrating information flow scores, data on regulatory relationships, and the expression profile in response to rapamycin treatment.

Using our framework, we identify the most relevant transcription factors and construct the *effective response network* of TOR, which is responsible for the observed transcriptional changes due to TOR inhibition. Our approach, unlike experimental methods, is not limited to specific aspects of cellular response. Rather, it predicts transcriptional changes, as well as post-translational modifications in response to TOR signaling. The resulting interaction map greatly enhances our understanding of the mechanisms underlying the aging process and helps identify novel targets for further investigation of anti-aging regimes. It also reveals potential network biomarkers for diagnoses and prognoses of age-related pathologies and identifies mechanisms for control of cellular aging processes through multi-targeted and combinatorial therapies [36,37].

## Results and discussion

### Computing information flow scores from TORC1

**Figure 1:**
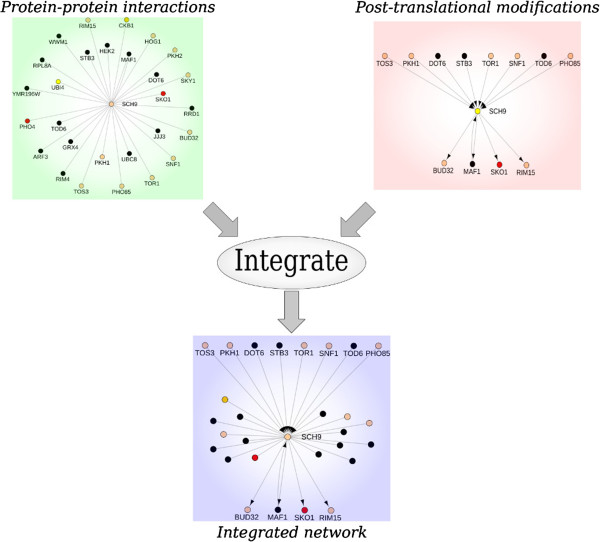
**Network integration process.** Example of the network integration process around Sch9p. Protein-protein interactions (PPI) and post-translational modifications (PTM) were extracted from BioGRID dataset.

**Figure 2:**
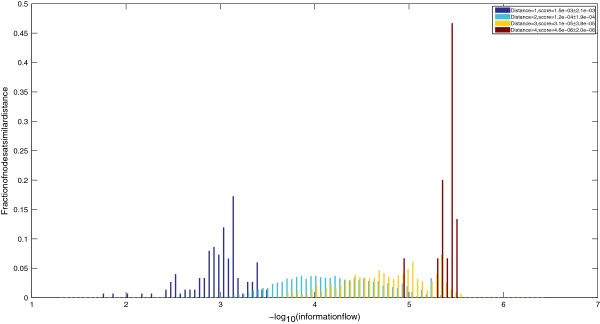
**Distribution of random-walk scores.** Information flow versus node distance from TORC1, showing that random-walk scores are a function of distance, as well as multiplicity of paths.

Given the yeast interactome, constructed using the procedure detailed in Methods Section and illustrated in Figure 1, we compute information flow scores using random walks initiated at selected nodes in the interactome. These nodes comprise members of the TORC1 complex, each of which propagates a unit flow (normalized to 0.2 for each of the five member proteins). We use a discrete random-walk process in which, at each step, every protein aggregates incoming signals and distributes them equally among outgoing neighbors. The final information flow scores are computed as the steady-state distribution of the random-walk process. One of the key parameters in the random-walk process, which controls the depth of propagation, is called the *restart-probability*. This is the probability that a random walker continues the walk (as opposed to teleporting to a node chosen from among a set of preferred nodes). In order to give all nodes in the interactome a chance of being visited, we use the relationship between restart probability and the mean depth of random-walks by setting parameter *α* to be equal to $\frac{d}{1+d}$, where *d* is the diameter of the interactome. For the yeast interactome, we determine the diameter to be equal to 6 and set $\alpha =\frac{6}{7}∼0.85$, correspondingly (please see the Methods section for details of information flow computations). Figure 2 illustrates the distribution of computed information flow scores, starting from TORC1, as a function of node distance from TORC1. It is evident from the figure that computed scores are functions of both distance from source nodes, as well as multiplicity of paths between source and sink nodes. This can be verified from the overlapping tails of distributions for nodes at different distances, as well as the varied distribution of scores among nodes at the same distance from TORC1. The final list of computed information flow scores is available for download as Additional file 1.

Node rankings from the random walk process are susceptible to degree-bias, favoring high-degree nodes. To remedy this bias and to gain a detailed mechanistic understanding of the roles of various proteins (and associated signaling elements), random walk methods need to be coupled with appropriate statistical tests. A key contribution of our work is the development of such a test, which yields a fine-grained understanding of key pathways involved in orchestrating cellular response to TOR inhibition. To the best of our knowledge, this work represents the first application of information flow methods for reconstructing the effective response network of TORC1.

### Constructing a high-level functional map of TOR downstream effectors

TORC1 is not only regulated by the quality and the quantity of both carbon and nitrogen sources [34,38-40], but also by noxious stressors, such as caffeine [41,42]. In response, TORC1 coordinately orchestrates various aspects of cellular machinery to mediate cell growth [32,40]. This includes autophagy [43], stress response [42,44], and protein synthesis (by regulating ribosome biogenesis [45], translation initiation [46], and nutrient uptake [47,48]).

In order to systematically identify the functional aspects relevant to TOR signaling, we first rank the proteins in the yeast interactome based on their information flow scores from the TORC1 complex. Given this ranked list, we aim to identify functional terms that are highly over-represented among top-ranked proteins. Gene Ontology (GO) [49] enrichment analysis has been used extensively for this purpose. We employ GOrilla [50] to find the optimal cut for each GO term, together with its exact minimum hypergeometric (mHG) p-value. Next, we enforce a threshold of *p*-value≤10^−3^ to identify the significant terms. The complete list of enriched terms for each branch of GO is available for download as Additional files 2.

**Figure 3:**
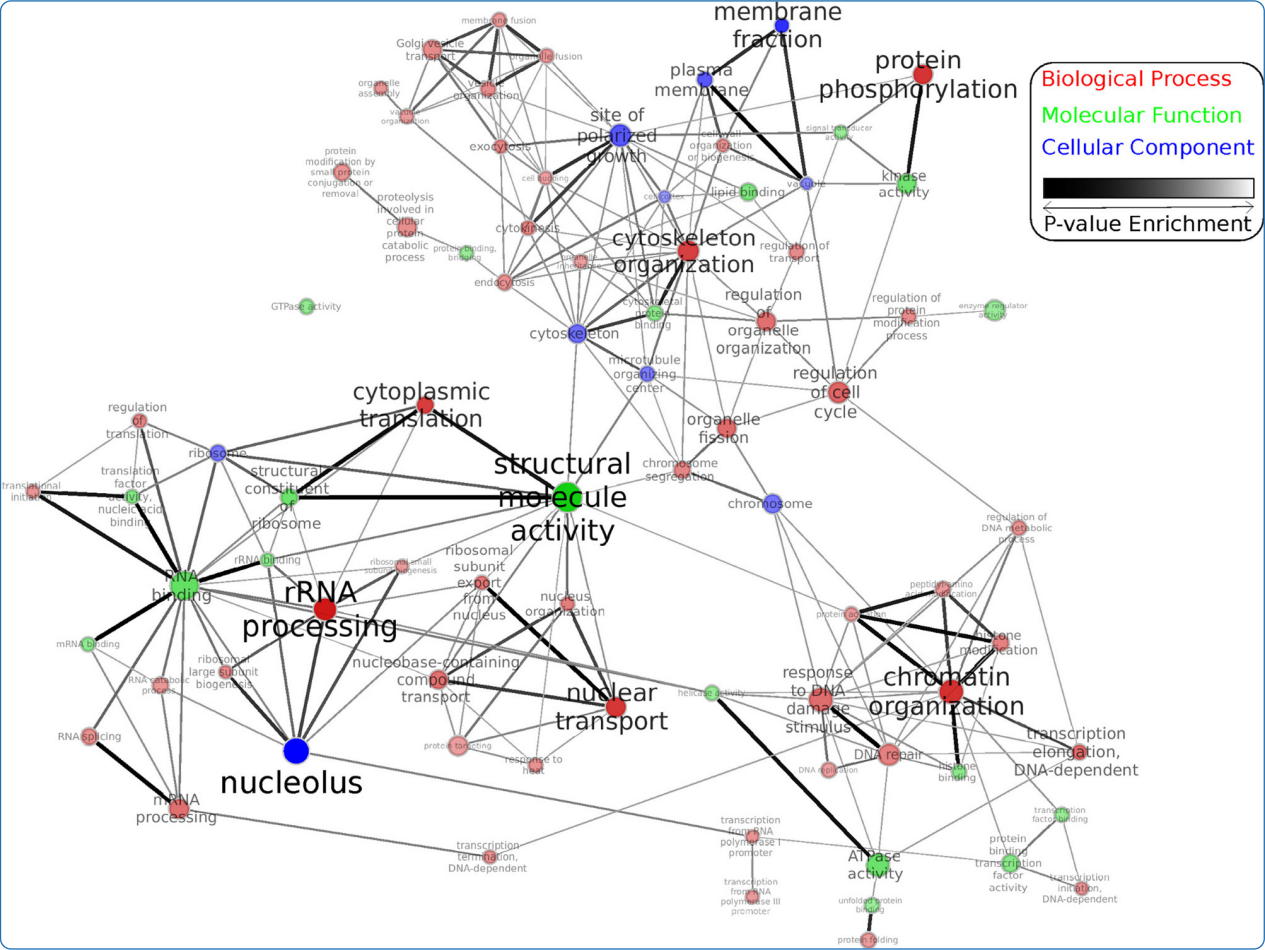
**Enrichment map of yeast GO slim terms.** Enriched terms are identified by mHG p-value, computed for the ranked-list of genes based on their information flow scores. Each node represents a significant GO term and edges represent the overlap between genesets of GO terms. Terms in different branches of GO are color-coded with red, green, and blue. Color intensity of each node represents the significance of its p-value, while the node size illustrates the size of its geneset. Thickness of edges is related to the extent of overlap among genesets.

GO provides a hierarchical vocabulary to annotate biological processes (BP), molecular functions (MF), and cellular components (CC). This hierarchical structure, represented using a directed acyclic graph (DAG), introduces an inherent dependency among the significant terms identified by GO enrichment analysis. Furthermore, seemingly independent terms under different branches of GO may be used to annotate the same set of genes. To provide a compact, non-redundant representation of the significant terms in our experiment, we follow a two-step process. First, we extract the subset of enriched terms that are marked by the Saccharomyces Genome Database (SGD) [51] as GO slim. Yeast GO slim is a compact subset of the entire GO, selected by SGD curators, which is necessary and sufficient to describe different aspects of yeast cellular biology. Next, we use EnrichmentMap (EM) [52], a recent Cytoscape [53] plugin, to construct the network (map) of the enriched terms. In this network, unlike the original interactome, each node represents a significant GO slim term and each weighted edge indicates the extent of overlap between genesets of their corresponding terms. We use a custom visualization style to illustrate various network properties. GO terms under BP, MF, and CC branches are color-coded red, green, and blue, respectively. The p-value of each term determines the opacity of both the node and its label; the bolder a term appears, the more significant its enrichment score. Finally, the total number of enriched genes for each GO term is shown using the size of the corresponding node. The final map, which is shown in Figure 3, is available for download as Additional file 3. This map provides unique opportunities for studying TOR-dependent terms visually, since terms (nodes) representing relevant sets of genes tend to cluster together in this network.

First, we note that most of the previously known targets of TORC1 are also identified by our information flow method, as represented in the enrichment map. For example, all terms related to *ribosome biogenesis*, including relevant cellular components (such as *ribosome* and *nucleolus*), molecular functions (such as *rRNA binding* and *structural constitute of ribosome*), and biological processes (such as *rRNA processing* and *ribosomal subunit export from nucleus*), are clustered in the bottom-left corner of the map. These terms, interestingly, are also clustered with other terms related to protein synthesis, such as *regulation of translation*, *translational initiation*, and *cytoplasmic translation*. Furthermore, many of the terms related to stress-response, such as *response to DNA damage stimulus* and *DNA repair*, are clustered in the bottom-left corner of the map. Finally, many of the terms related to TOR signaling, nutrient uptake, and cytoskeleton organization are grouped on the top section of the map.

**Figure 4:**
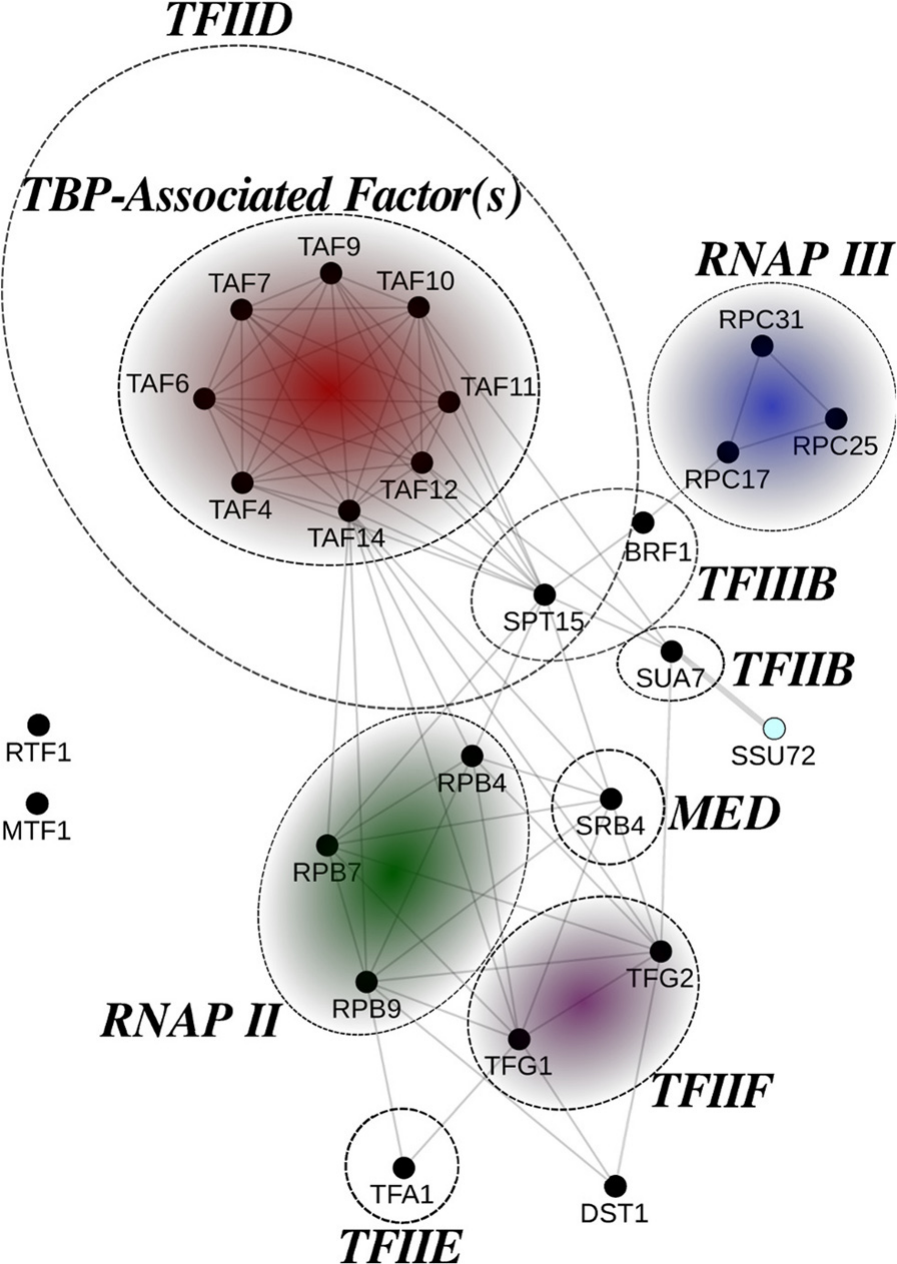
**TOR-dependent control of transcription initiation.** Induced subgraph in the yeast interactome, constructed from the top-ranked genes in the information flow analysis that are annotated with the transcription initiation GO term. Different functional subunits are marked and color-coded appropriately.

Additionally, we observe that there are terms in this map that have not been adequately investigated in previous efforts. For example, even though translational control is a well-known function of TORC1, transcriptional control is less-studied. Several terms related to transcription initiation and elongation are enriched in our analysis, as shown on the bottom-right of the map. In order to gain a mechanistic understanding of these terms, we project the geneset of each term (node) back to the original network and construct the corresponding induced subgraph in the yeast interactome. As a case study, we extract the set of enriched genes represented by the *transcription initiation* GO term and construct its induced subgraph, which is shown in Figure 4. Here, nodes, representing proteins, are grouped and annotated based on their functional role in forming the transcription pre-initiation complex (PIC), as well as the RNA polymerase (RNAP). The basal level of transcription in Eukaryotic cells by RNAP needs a family of general transcription factors (GTF), prior to the formation of PIC. The TATA-binding protein (TBP), encoded by the Spt15 gene in yeast, is a universal GTF that is involved in transcription by all three types of nuclear RNAP. As a component of TFIIIB complex, it forms the PIC complex and recruits RNAPIII to the transcriptional start site(TSS) of tRNAs, 5S rRNA, and most snRNAs. As a part of TFIID, it forms a complex together with TBP-associated factors (TAF) and binds to the core promoter region of the protein-coding genes, as well as some snRNAs. The correct assembly of PIC, required for directing RNAPII to the TSS, needs additional GTFs, namely TFIIA, -B, -D, -E, -F, and TFIIH, as well as the Mediator (MED) complex. These components are assembled in an orderly fashion to form the PIC and mediate the transcription initiation by RNAPII (please see Hampsey [54] and Maston et al. [55] for a review). These complex interactions are faithfully reconstructed in Figure 4, which provides a more refined understanding of transcription initiation, under TOR control, in the yeast cells.

### Comparison of predicted targets to the set of differentially expressed genes in response to Rapamycin treatment

Rapamycin, a lipophilic macrolide originally purified as an antifungal agent and then re-discovered as an immunosuppressive drug, forms a toxic complex with its intracellular receptor FKBP12, encoded by the Fpr1 gene in yeast, and directly binds to TOR in order to perform its inhibitory action [32]. We hypothesize that if the information flow-based method agrees with the TORC1 signaling network, it should be able to predict transcriptional changes due to rapamycin treatment, which inhibits TORC1 *in vivo*. To validate this hypothesis, we used a recent mRNA expression profile of yeast in response to rapamycin treatment [56]. We extracted the set of differentially expressed genes, at a minimum threshold of 2-fold change, and constructed a vector of true positives from this set by filtering out genes that do not have a corresponding vertex in the yeast interactome. The final dataset includes 342 repressed and 237 induced genes in our experiment.

**Figure 5:**
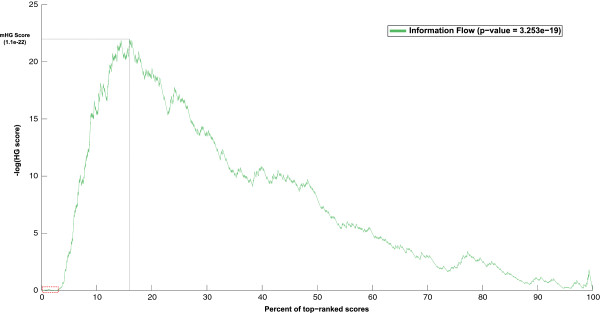
**Enrichment plot for rapamycin-treatment dataset.** Enrichment score as a function of the score percentage. Computations are based on the set of differentially expressed genes in response to rapamycin treatment. The peak of plot occurs at around top 15% of scores, with the corresponding exact p-value of 3.3*10-19.

Using this set of true-positives, we computed the *enrichment plot* of information flow scores by ranking all proteins and computing the hypergeometric score as a function of the protein rank, which is illustrated in Figure 5. The peak of the plot, corresponding to the minimum hypergeometric (mHG) score, occurs at the index *l*=906 from the top, which covers approximately the top 15% of scores. There are 181 positive genes in this partition, from a total of 579 positives, yielding a mHG score of 1.11∗10^−22^. We computed the exact p-value corresponding to this mHG score, using the dynamic-programming method of Eden et al. [57], resulting in the significant enrichment p-value of 3.25 ∗ 10^−19^. This in turn supports our hypothesis that the random-walk neighborhood of TORC1 is highly enriched with the set of genes that are differentially expressed in response to rapamycin treatment.

### Post-translational modifications among top-ranked proteins: a case study on Gap1 regulation

An interesting observation from Figure 5 is that the highest-ranked genes (approximately the top 150 genes), marked with a red box, are not enriched in terms of rapamycin-induced genes. This can be explained by the fact that regulatory elements in the TOR signaling pathway, including TFs, do not typically change their expression level in response to TOR signaling. Instead, they are targeted for post-translational modifications (typically, phosphorylation). We consequently hypothesize that the top genes should also be enriched in terms of phosphorylation events. To further investigate this hypothesis, we focus on a case study of *Gap1* regulation, a general amino acid permease regulated by NCR. We choose Gap1 since its regulatory pathway, originating from TORC1, is well-studied in literature. Moreover, data from phosphoproteomic experiments, which measures phosphorylation events among elements of this pathway, is readily available. Specifically, Gap1 is positively regulated via Gln3 and Gat1, while it is repressed by Gzf3 and Dal80 [34,40]. Interestingly, all four of these regulators are among top-ranked transcription factors, yet none of them are differentially expressed in response to rapamycin treatment. Using a recent phosphoproteome of yeast in response to rapamycin treatment [58], we validated that both of the transcriptional activators of Gap1, namely *Gln3* and *Gat1*, are highly phosphorylated in response to rapamycin treatment. Moreover, Tap42-Sit4, which is the upstream regulator of Gcn4, is indirectly regulated by TORC1.

**Figure 6:**
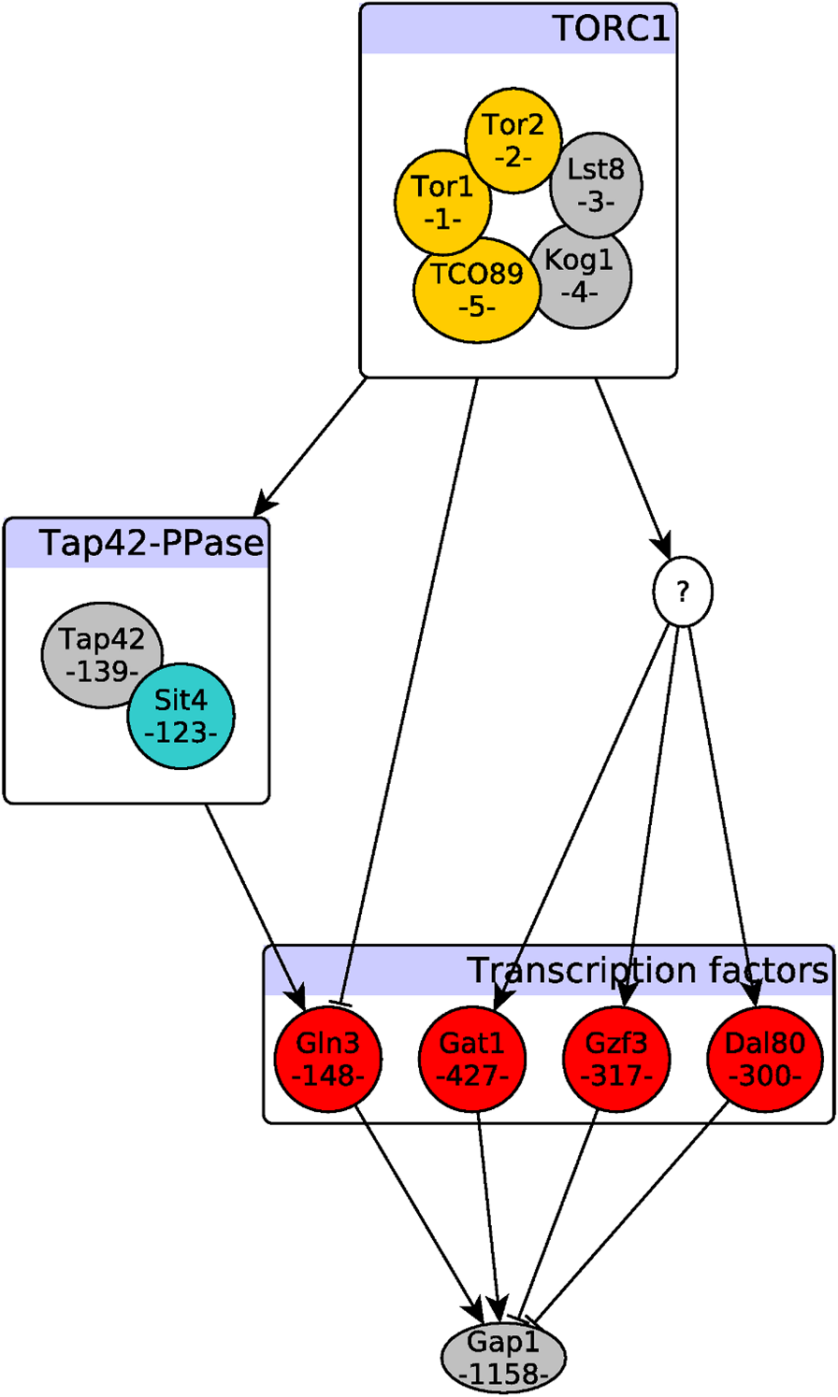
**TORC1-dependent regulation of Gap1.** The schematic diagram is based on literature evidence for the known interactions. Each node in the signaling pathway is annotated with the rank of its information flow score from TORC1 and colored with its functional classification. Yellow nodes represent kinase associated proteins, red nodes are transcription factors, and blue node (Sit4) is a phosphatase. The rest of nodes have a default color of grey. Ranking of nodes based on their information flow scores coincides with our prior knowledge on the structure of this pathway. Top/bottom ranked nodes are discriminated using the computed cutoff value (*l*) based on differentially expressed genes. The “?” indicates an unknown underlying mechanism, yet to be discovered, that connects TORC1 to the rest of transcription factors.

Figure 6 illustrates this signaling pathway, with each element annotated using its information flow rank. All signaling elements upstream of Gap1 are present among top-ranked scores, yet none of them change their expression levels in response to rapamycin treatment. This partially supports our hypothesis that the top-ranked genes in the random-walk are primarily targets of post-translational modifications. However, a more thorough experimental analysis of the the top-ranked proteins potentially may reveal currently unknown mechanisms by which yeast cells respond to TOR signaling. To this end, our computational studies motivate and provide data for future experimental investigations.

### Sensitivity and specificity of information flow scores in predicting key transcription factors

Top-ranked proteins in information flow analysis are highly enriched in terms of differentially expressed genes under rapamycin treatment. However, TORC1 does not directly regulate expression of these genes. This observation raises the question: which transcription factors are responsible and which intermediary elements are involved in these regulations? We answer the first question here, while deferring the latter to subsequent sections.

To find the key transcription factors that modulate the observed transcriptional response, we use two separate statistical predictors, one based on the information flow scores and the other based on the set of differentially expressed genes. These predictors allow us to assess the significance of TFs with respect to their computationally computed, top-ranked and experimentally validated targets, respectively. In the first method, we call a transcription factor *relevant* if a significant fraction of its target genes are highly-ranked in information flow method. Conversely, in the second method we define the relevance in terms of the portion of its differentially expressed targets (please see Equations 6-8 for details).

We use *p*-value(*X*=*k*_*T*_) and *p*-value(*Y*=*k*_*P*_) and apply a cutoff value of *ε*=0.01 to identify significant p-values computed for computational and experimental predictions, respectively. At this threshold, we compute the sensitivity and specificity of information flow methods as 0.2245 and 0.9846, respectively. The observed high specificity value suggests that if targets of a given TF are not differentially expressed, with high probability, our computational model also reports it as a negative (it will not have significant number of top-ranked targets). In other words, transcription factors that are identified as significant using information flow scores are highly precise. On the other hand, the lower sensitivity score implies that even if a TF has many differentially expressed targets, our computational method may miss it. From this, we can conclude that transcription factors that have significant numbers of top-ranked targets are high-confidence candidate(s) as downstream effectors of TORC1. However, there are cases where we may miss relevant transcription factors with a significant number of differentially expressed genes by this approach. In the next section, we propose a statistical framework to integrate information flow scores and expression profiles to reliably identify the most relevant subset of transcription factors that are involved in mediating the transcriptional response to TOR inhibition, and consequently construct the effective response network of TORC1.

### Identifying the most relevant transcription factors

We now seek to integrate experimental measurements from rapamycin treatment, information flow scores, and the transcription regulatory network into a unified framework to identify the most relevant transcription factors. To this end, we introduce the notion of *relevance score*. Let random variable *Z* denote the number of top-ranked positive targets, and *k*_*TP*_ denote the number of top-ranked positive targets of a given TF. We define the relevance score as − log(*p*-value(*Z*=*k*_*TP*_)). The relevance score assesses both positivity and rank of the targets for a given TF (please see Equation 10 for details). Using this approach, we identify 17 TFs with high relevance scores, which are hypothesized to be responsible for the transcriptional changes in a TORC1-dependent manner. The complete list of computed statistics for all transcription factors is summarized in Additional file 4.

The top five transcription factors are listed in Table 1. Among these top-ranked, high confidence, transcription factors, Sfp1, Gln3, and Gcn4 are well-documented downstream effectors of TORC1 [48,59-61] (please see Zaman et al. [34], Smets et al. [40], and Loewith and Hall [32] for a more comprehensive review). *Sfp1* is a stress- and nutrient-sensitive regulator of cell growth, responsible for mediating the expression of genes involved in ribosome biogenesis, such as RP genes and RiBi factors [62,63]. TORC1 mediates Sfp1-related genes by phosphorylating Sfp1 and regulating its nuclear localization [59]. *Gln3*, a GATA-family transcription factor, positively regulates the expression of nitrogen catabolite repression (NCR)-sensitive genes [60,64]. TORC1-dependent regulation of Gln3 is mediated by promoting its association with its cytoplasmic anchor protein Ure2 [32,65]. *Gcn4* is a nutrient-responsive transcription factor, which is activated upon amino acid starvation [66]. TORC1 regulates Gcn4 by mediating its translation level in a eIF2 *α*-dependent manner [32]. Interestingly, Steffen et al. [61] also proposed a critical role for Gcn4 in mediating life-span in yeast.

**Table 1:** Top-ranked transcription factors with high confidence scores

**TF ORF**	**TF name**	**TF rank**	**TF confidence**
YLR403W	SFP1	22	43.5048
YER040W	GLN3	148	57.7734
YML007W	YAP1	618	24.3672
YEL009C	GCN4	638	4.822
YHR084W	STE12	825	2.9668

However, to the best of our knowledge, Ste12 and Yap1 have not been previously positioned downstream of TORC1. Ste12 is best known as a downstream target of mitogen-activated protein kinase (MAPK) signaling cascade and is responsible for regulating genes involved in mating or pseudohyphal/invasive growth [67]. Rutherford et al. [68] show that over-expression of the ammonium permease Mep2 induces the transcription of known targets of Ste12. A more recent study by Santos et al. [69] additionally positions TORC1 downstream of Mep2, which, taken together with the link between Mep2-Ste12, suggests Ste12 as a potential downstream effector of TORC1. *Yap1* is an AP-1 family transcription factor required for inducing oxidative [70,71] and carbon [72] stress responses, the latter is proposed to be independent of TORC1. Additionally, Yap1 expression has been shown to increase significantly during replicative aging [73]. It has been suggested that spermidine, a conserved longevity factor [74], mediates macroautophagy in a Yap1 and Gcn4 dependent manner [75]. Finally, there is a diverse set of age-related functions associated with Yap1, many of which are also attributed to TORC1. These observations suggest Yap1 as a potential candidate downstream effector of TORC1.

### Constructing the effective response network of TORC1

**Figure 7:**
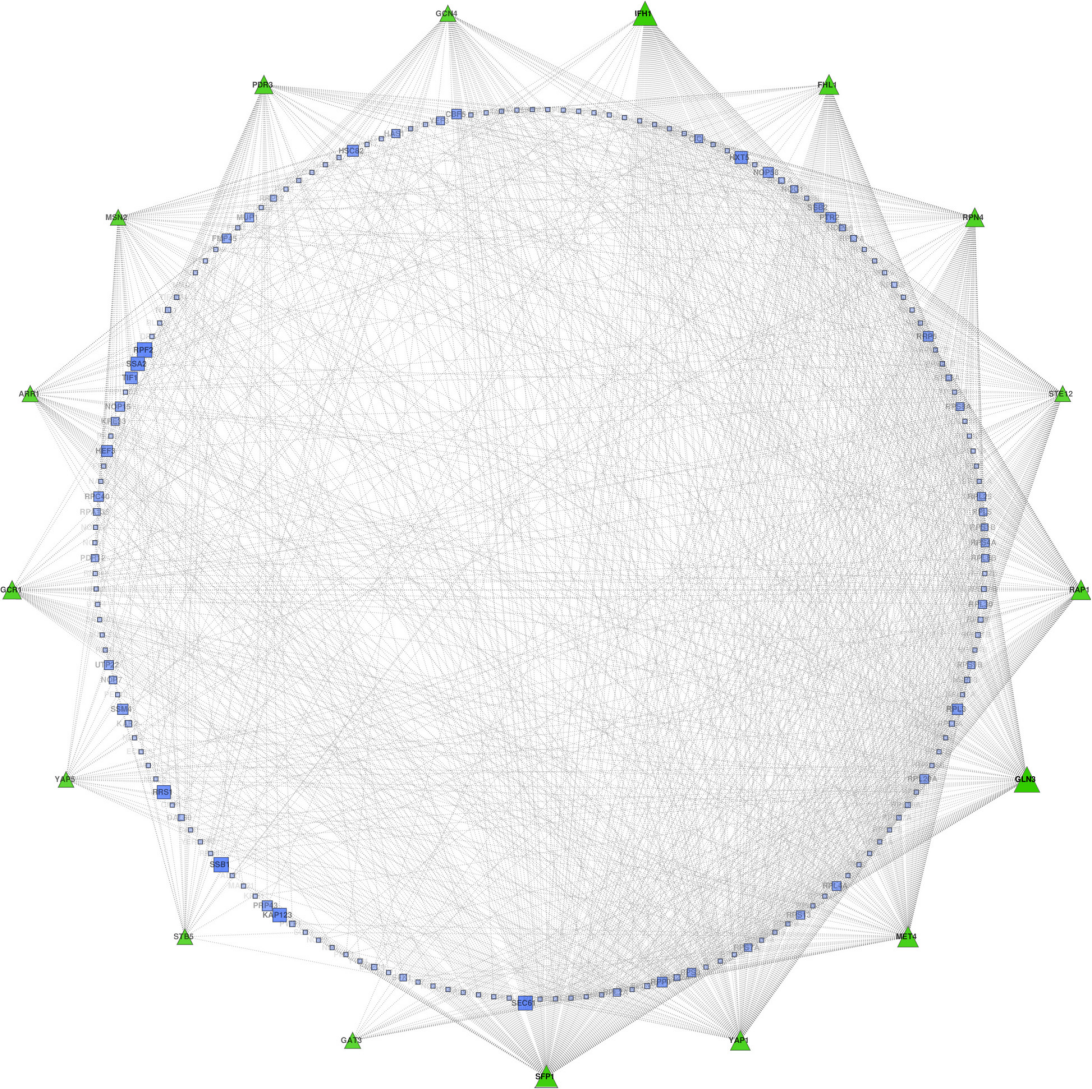
**Effective response network (ERN) of TORC1.** The effective response network is computed for most relevant transcription factors, yielding a network of 1,288 transcription regulations between 17 TFs and 181 target genes. Green nodes represent TFs while blue nodes are the target genes. The size and and color intensity of TFs and target genes represent their relevance score and information flow score, respectively.

To uncover the regulatory mechanisms that mediate the response to TOR inhibition, we construct the *effective response network (ERN)* of TORC1, which is illustrated in Figure 7 and is available for download as Additional file 5. Node attributes for this network are available for download separately as Additional file 6. This network consists of the most relevant TFs, together with their top-ranked positive targets, with a total of 1,288 regulatory interactions between 17 transcription factors and 181 target genes.

**Figure 8:**
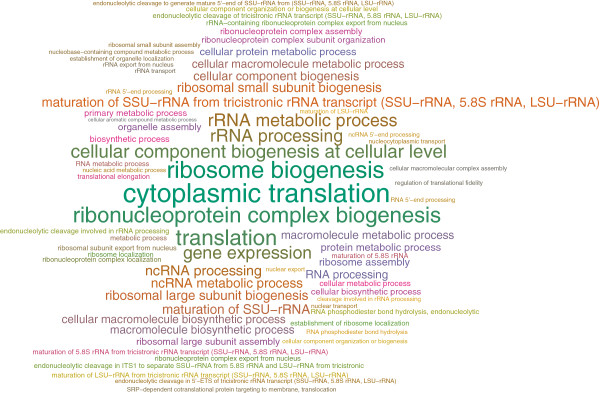
**Enrichment analysis of the ERN.** Static word cloud for the enriched BP terms in the effective response network (ERN).

In order to better understand the functional roles of the predicted targets, we use FIDEA [76] to identify enriched GO terms under the biological process (BP) branch. Figure 8 illustrates the static word cloud of the enriched terms, as generated by FIDEA, the complete list of which is available for download as Additional file 7. Unlike the enrichment map of TORC1, which spans a variety of different functions, targets in the effective response network (ERN) are almost exclusively involved in ribosome biogenesis and the cellular translation process. Ribosome biogenesis is one of the most energy-consuming tasks in the cell that is directly linked to the rate of translation and is required for cell growth [77]. Calorie restriction, or alternatively inhibiting TORC1 by Rapamycin treatment, is known to coordinately regulate this process via a complex set of pathways involving transcription factors *Ifh1, Sfp1, Fhl1*, and *Rap1*[77]. Interestingly, all four of these transcription factors are identified by our method among the top 6 TFs with the highest relevance scores (together with *Gcn3* and *Met4*). The effective response network provides a refined view of how yeast cells re-wire various aspects of ribosome biogenesis in order to modulate cell growth. This network can be used to gain a detailed understanding of the regulatory mechanisms that are responsible for TOR-dependent transcriptional changes in yeast.

## Conclusions

Understanding various processes associated with aging has important implications for the diagnosis, prognosis, and treatment of age-related pathologies. Current methods for constructing aging pathways rely on detailed experiments that study cellular response to carefully controlled signals. This process is expensive, time-consuming, and typically restricted to specific aspects of cellular response. In this study, we presented a complementary, computational approach that aims to construct detailed aging pathways using the yeast interactome by initiating random walks at proteins that are key players in the aging process (the target of rapamycin or TOR, in this study). At the heart of our method is a rigorous statistical and computational framework that identifies significant effector proteins and provides information about the specific mechanisms associated with them.

We present comprehensive validation of our computational results through GO enrichment studies and manual curation to show that our method identifies most of the known proteins downstream from TOR, while identifying several new proteins for future experimental investigations. Additionally, we showed that information flow scores faithfully predict transcriptional changes in response to rapamycin-treatment, which validates accuracy of predicted effectors. Furthermore, we show that the predicted targets are also enriched with proteins that are post-translationally modified (i.e., phosphorylated) in response to TOR inhibition. Finally, we constructed the *effective response network* of the TOR pathway. This network is hypothesized to mediate transcriptional changes in response to TOR inhibition. A direct outcome of our study is a complete dataset of proteins downstream of TOR, their interactions, functional roles, and organization.

## Methods

### Constructing yeast interactome

We obtained the yeast protein-protein interactions (PPI) from the BioGRID [78] database, update 2011 [79], version 3.1.83, by extracting all physical interactions, except for protein-RNA interactions, and excluding interspecies and self interactions. This dataset consists of 103,619 (63,395 non-redundant) physical interactions among 5,691 proteins, and is available for download as Additional file 8. We then identified the subset of interactions associated with post-translational modification (PTM), marked with the “biochemical activity” evidence code in BioGRID, resulting in 5,791 (5,443 non-redundant) biochemical activities among proteins in yeast. These are available for download as Additional file 9. Each of these interactions represents a directional enzymatic activity, where the *bait* protein executes the activity on the substrate *hit* protein. After integrating different modifications among similar pairs of proteins, we obtained 5,421 directional edges among 2,002 proteins in the yeast interactome. The bulk of these interactions (over 4,000) are the phosphorylation events identified by Ptacek et al. [80] using proteome chip technology.

We constructed the integrated network of yeast interactions, the *yeast interactome*, by integrating protein-protein interactions(PPIs) and post-translational modifications(PTMs). For pairs of proteins that have both PPI and PTM, we give higher priority to PTM, since it provides a more refined description of the type of interaction. Figure 1 illustrates an example of the integration process around the *Sch9* protein, which is a well-documented substrate of TORC1. The final constructed interactome is available for download as Additional file 10. This network consists of 5,287 uni-directional and 58,108 bi-directional edges (58,041 PPIs and 134 bi-directional PTMs) among 5,691 nodes. The node attributes and alternative labels for each node in the yeast interactome are also available for download as Additional file 11.

### Transcriptional regulatory network (TRN) of yeast

We constructed the yeast transcriptional regulatory network (TRN) from the documented regulations in YEASTRACT [81], consisting of 48,082 interactions between 183 transcription factors (TF) and 6,403 target genes (TG). Among these 183 TFs, 179 of them have a corresponding node in the yeast interactome.

### Tracing information flow in the interactome

We use a computational approach, based on a discrete-time random walk process, to track directional information flow in the interactome. Similar formulations have been previously used to prioritize candidate disease genes [82,83], discover network bio-markers for cancer [84], and identify protein complexes [85,86]. Additionally, there is a known correspondence between random-walk methods on undirected graphs and formulations based on circuit network models [87]. Our formulation takes into account both network distances, as well as multiplicity of paths between pairs of proteins. It also benefits from using edge directions (when available) to discriminate between upstream regulators and downstream effectors.

Let *G*=(*V*,*E*) be a mixed graph, having both directed and undirected edges. Each node in *V* corresponds to a protein and edge (*u*,*v*)∈*E* iff protein *u* interacts with protein *v* in the integrated network. Graph *G* can be represented using its adjacency matrix *A*, where *A*_*ij*_=1, if node *i* has a directed edge to node *j*, and is 0 otherwise. Undirected edges are replaced by a pair of directed edges in each direction. A *random walk* on *G*, initiated from vertex *v*, is defined as a sequence of transitions among vertices, starting from *v*. At each step, the random walker randomly chooses the next vertex from among the neighbors of the current node. The sequence of visited vertices generated by this random process is a Markov chain, since the choice of next vertex depends only on the current node. We can represent the transition matrix of this Markov process as a column-stochastic matrix, *P*, where *p*_*ij*_=*Pr*(*S*_*t*+1_=*v*_*i*_|*S*_*t*_=*v*_*j*_), and random variable *S*_*t*_ represents the state of the random walk at the time step *t*.

*Random walk with restart (RWR)* is a modified Markov chain in which, at each step, a random walker has the choice of either continuing along its path, with probability *α*, or jump (teleport) back to the initial vertex, with probability 1−*α*. Given the transition matrix of the original random walk process, *P*, the transition matrix of the modified chain, *M*, can be computed as *M* = *αP*+(1−*α*)***e***_*v*_***1***^*T*^, where ***e***_*v*_ is a stochastic vector of size *n* having zeros everywhere, except at index *v*, and ***1*** is a vector of all ones. The stationary distribution of the modified chain, ***π***_*v*_(*α*), defines the portion of time spent on each node in an infinite random walk with restart initiated at node *v*, with parameter *α*. This stationary distribution can be computed as follows:

(1) $$\begin{array}{l} \pi_{v}(\alpha )=M\pi_{v}(\alpha )\\ \;=(\mathrm{\alpha P}+(1-\alpha )e_{v}1^{T})\pi_{v}(\alpha ) \end{array}$$

Enforcing a unit norm on the dominant eigenvector to ensure its stochastic property, ∥***π***_*v*_(*α*)∥_1_=***1***^*T*^***π***_*v*_=1, we will have the following iterative form:

(2) $$\pi_{v}(\alpha )=\mathrm{\alpha P}\pi_{v}(\alpha )+(1-\alpha )e_{v},$$

which is a special case of the personalized PageRank [88-91], with preference vector ***e***_*v*_. Alternatively, we can compute ***π***_*v*_ directly by solving the following linear system:

(3) $$\pi_{v}(\alpha )=\underset{Q}{\underset{︸}{(1-\alpha ){\left(I-\mathrm{\alpha P}\right)}^{-1}}}e_{v},$$

where the right-multiplication with ***e***_*v*_ simply selects column *v* of the matrix *Q*. The factor 1−*α* can be viewed as the *decay factor* of the signal; the higher the parameter *α*, the further the signal can propagate. Let us denote by random variable *R* the number of hops taken by random walker before it jumps back to source node *v*. Then, *R* follows a geometric distribution with probability of success (1−*α*) and the expected (mean) length of paths taken by random walker can be computed as $E(R)=\frac{\alpha }{1-\alpha }$. In other words, if we let $\alpha =\frac{d}{1+d}$, for a given value of *d*, we expect the average length of paths taken by such a random walk to be equivalent to *d*, thus we call *d* the depth of the random walk.

### Cross-validating information flow scores with the set of differentially expressed genes in response to TOR inhibition

Given the list of gene products ranked by their information flow scores, we want to assess the enrichment of differentially expressed genes, in response to rapamycin treatment, among top-ranked proteins.

The classical approach to this problem is to select a predefined cutoff on ranks, denoted by *l*, which separates the top-ranked genes (target set) from the rest (background set), and then compute the enrichment p-value using the hypergeometric distribution. Let us denote the total number of gene products by *N* and the total number of differentially expressed genes (true positives) by *A*. Using a similar notation as Eden et al. [57], we encode these annotations using a binary vector, *λ*=*λ*_1_,*λ*_2_,…*λ*_*N*_∈{0,1}^*N*^, having exactly *A* ones and *N*−*A* zeros. Let the random variable *T* denote the number of positive genes in the target set, if we distribute genes randomly. In this formulation, the hypergeometric p-value is defined as:

(4) $$\begin{array}{l} p\text{-value}(T=b_{l}(\lambda ))=\text{Prob}(b_{l}(\lambda )\leq T)\\ \;=\text{HGT}(b_{l}(\lambda )|N,A,l)\\ \;=\overset{\text{min}(A,l)}{\underset{t=b_{l}(\lambda )}{\sum }}\frac{C(A,t)C(N-A,l-t)}{C(N,l)}, \end{array}$$

where *HGT* is the tail of hypergeometric distribution, and $b_{l}(\lambda )=\sum_{i=1}^{l}\lambda_{i}$, is the number of observed positives in the target set. The drawback of this approach is that we need a predefined cutoff value, *l*. To remedy this, Eden et al. [57] propose a two-step method for computing the exact enrichment p-value, called *mHG p-value*, without the need for a predefined cutoff value of *l*. In the first step of this process, we identify an optimal cut, over all possible cuts, which minimizes the hypergeometric score. The value computed in this manner is called the *minimum hypergeometric (mHG) score*, and is defined as:

(5) $$\text{mHG}(\lambda )={\text{min}}_{1\leq l\leq N}\text{HGT}(b_{l}(\lambda )|N,A,l)$$

Next, we use a dynamic programming (DP) method to compute the exact p-value of the observed mHG score, in the state space of all possible *λ* vectors with size *N* having exactly *A* ones (please refer to Eden et al. [57] for algorithmic details, and Eden [92] for an efficient implementation). We adopt this strategy to cross-validate our results with the transcriptome profile of yeast cells in response to rapamycin treatment. We subsequently define the *enrichment plot*, which illustrates the absolute value of the logarithm of the HG score as a function of cutoff percentage. The minimum hypergeometric (mHG) score can be viewed as the peak of this plot, and the corresponding exact p-value can be computed for this peak using the aforementioned DP algorithm.

### Assessing the sensitivity and the specificity of information flow scores

Given an optimal cutoff length *l* (computed for differentially expressed genes in response to TOR inhibition), which partitions nodes into top/bottom ranked proteins, together with a transcription factor (TF) of interest, *p*_*i*_, we are interested in assessing the importance of *p*_*i*_ in mediating the observed transcriptional response. In other words, given that *p*_*i*_ has a significant number of top-ranked targets, how confident are we that it will also have a significant number of differentially expressed targets? Conversely, if *p*_*i*_ has many differentially expressed targets, how likely is it to see its targets among top-ranked genes?

Let us denote the total number of targets of TF *p*_*i*_ by *k*, and the number of its positive (differentially expressed) and top-ranked (in information flow) targets by *k*_*P*_ and *k*_*T*_, respectively. Let the random variable *X* be the number of top-ranked targets, if we were uniformly distributing *k* targets of *p*_*i*_ among all genes in the yeast interactome. Similarly, let *Y* be the number of positive targets of *p*_*i*_, if we distribute positive targets uniformly. Then, we can compute the following p-values for top-ranked and positive targets, respectively:

(6) $$\begin{array}{l} p\text{-value}(X=k_{T})=\text{Prob}(k_{T}\leq X)\\ \;=\text{HGT}(k_{T}|N,l,k)\\ \;=\overset{\text{min}(l,k)}{\underset{x=k_{T}}{\sum }}\frac{C(l,x)C(N-l,k-x)}{C(N,k)} \end{array}$$

(7) $$\begin{array}{l} p\text{-value}(Y=k_{P})=\text{Prob}(k_{P}\leq Y)\\ \;=\text{HGT}(k_{P}|N,A,k)\\ \;=\overset{\text{min}(A,k)}{\underset{y=k_{P}}{\sum }}\frac{C(A,y)C(N-A,k-y)}{C(N,k)} \end{array}$$

After correcting for multiple hypothesis testing using Bonferroni method, we use a given threshold value of *ε* and define the *sensitivity* and *specificity* for the entire set of transcription factors as:

(8) $$\begin{array}{l} \text{sensitivity}=\text{Prob}(p\text{-value}(X=k_{T})\\ \;\leq \varepsilon |p\text{-value}(Y=k_{P})\leq \varepsilon )\\ \text{specificity}=\text{Prob}(\varepsilon <p\text{-value}(X=k_{T})|\varepsilon \\ \;<p\text{-value}(Y=k_{P})) \end{array}$$

The motivation behind our approach is that the set of transcription factors with a significant number of differentially expressed targets provides us with an experimentally validated set of key factors, whereas transcription factors that have a significant number of top-ranked targets act as computational predictions for identifying the most relevant TFs. Let *TP* be the number of identified true positives, *P* be the total number of positives, and *FN* be the number of false negatives. The sensitivity of a method, defined as $\frac{\text{TP}}{P}=\frac{\text{TP}}{\text{TP}+\text{FN}}$, measures the fraction of positive instances (transcription factors having a significant number of differentially expressed targets) that are also predicted using the information flow method (computational predictions). Conversely, let *TN* be the number of true negatives identified by the method and *N* be the total number of negatives. Specificity, formally defined as $\frac{\text{TN}}{N}=\frac{\text{TN}}{\text{TN}+\text{FP}}$, corresponds to the fraction of irrelevant TFs, computed based on the experimental dataset, that are also identified as irrelevant by our computational predictions. These two measures are closely related to type I and II errors as follows:

(9) $$\begin{array}{c} \text{Type I error}(\alpha )=\text{False-positive-rate}(\text{FPR})\\ \;=1-\text{specificity}\\ \text{Type II error}(\beta )=\text{False-negative-rate}(\text{FNR})\\ \;=1-\text{sensitivity} \end{array}$$

### Integrating computational predictions with experimental datasets

Given the set of differentially expressed genes in response to rapamycin treatment, the computed information flow scores, and the transcriptional regulatory network (TRN) of yeast, we aim to construct an integrative statistical framework to identify the most relevant transcription factors with respect to mediating the transcriptional response to TOR inhibition, and decipher the underlying effective response network.

Let us denote the number of top-ranked positive targets of a given TF by *k*_*TP*_. If we compute the probability of observing *k*_*TP*_ or more positive targets among top-ranked genes, entirely by chance, we can subsequently identify the associated subset of relevant transcription factors. Let the random variable *Z* denote the number of top-ranked positive targets, if we were randomly distributing all targets for the given TF. We can compute the p-value of *Z* by conditioning it on the number of top-ranked targets as follows:

(10) $$\;\begin{array}{l} p\text{-value}(Z=k_{\text{TP}})=\text{Prob}(k_{\text{TP}}\leq Z)\\ \;=\overset{\text{min}(l,k)}{\underset{x=k_{\text{TP}}}{\sum }}\text{Prob}(k_{\text{TP}}\leq Z|X=x)\\ \;\times \text{Prob}(X=x)\\ \;=\overset{\text{min}(l,k)}{\underset{x=k_{\text{TP}}}{\sum }}\overset{\text{min}(x,b_{l}(\lambda ))}{\underset{z=k_{\text{TP}}}{\sum }}\text{Prob}(Z=z|X=x)\\ \;\times \text{Prob}(X=x)\\ \;=\overset{\text{min}(l,k)}{\underset{x=k_{\text{TP}}}{\sum }}\text{HG}(x|N,l,k)\\ \;\times \overset{\text{min}(x,b_{l}(\lambda ))}{\underset{z=k_{\text{TP}}}{\sum }}\text{HG}(z|l,b_{l}(\lambda ),x) \end{array}$$

After correcting for multiple hypothesis testing using Bonferroni method, we define the *relevance score* of each TF as − log10(*p*-value(*Z* = *k*_*TP*_)), and construct the effective response network of TORC1 using the most relevant TFs, together with their top-ranked positive targets, correspondingly.

## Availability of supporting data

The data sets supporting the results of this article are included within the article (and its additional files). They are also available for download from http://compbio.soihub.org/projects/torc1. All necessary codes and datasets to reproduce the results in this paper are bundled as Additional file 12.

## Abbreviations

TOR: Target of rapamycin; PPI: Protein-Protein Interaction.

## Competing interests

The authors declare that they have no competing interests.

## Authors’ contributions

SM conceived the study, designed and implemented methods, performed the experiments, and prepared the manuscript. SS helped with the experimental design, as well as analyzing and interpreting the biological implications of the results. AG provided guidance relative to the theoretical and practical aspects of the algorithms, and design of proper statistical model(s) to validate the results. All authors participated in designing the structure and organization of final manuscript. All authors read and approved the final manuscript.

## Supplementary Material

Additional file 1 **Information flow scores.** Excel table file (*.xls) illustrating the computed information flow scores for different proteins, sorted based on their proximity to TORC1. Click here for file

Additional file 2 **GO Enrichment of information flow scores.** Excel table files (*.zip) illustrating the GO enrichment analysis using mHG under BP, CC, and MF branches, respectively. Click here for file

Additional file 3 **Enrichment map of the significant GO terms.** Cytoscape file (*.zip) containing the final enrichment map. Each node represents a GO term and the corresponding geneset and enrichment p-value are encoded in its attributes. Edges are computed based on the geneset overlap among GO terms (please see http://www.cytoscape.org for more information about loading the file). Click here for file

Additional file 4 **Statistical analysis of the most relevant transcription factors (TF).** Excel table file (*.xls) illustrating the computed statistics for different transcription factors, sorted based on their proximity to TORC1. Click here for file

Additional file 5 **Effective response network (ERN) of TORC1.** Tab separated file (*.txt) representing the edge list of the effective response network of TORC1 (corresponding nodes are marked by their systematic name). Click here for file

Additional file 6 **Node attributes for the effective response network.** Tab-separated table file (*.txt) file containing additional attributes for the nodes in the ERN. Click here for file

Additional file 7 **Functional enrichment of the targets in the effective response network.** Tab-separated table file (*.txt) file containing the enriched BP terms in the ERN, identified by FIDEA. Click here for file

Additional file 8 **Yeast protein-protein interactions.** Tab-separated table file (*.txt) containing the non-redundant PPI edge list (corresponding nodes are represented using their Entrez Gene ID). Click here for file

Additional file 9 **Yeast biochemical activities.** Tab-separated table file (*.txt) containing the list of non-redundant PTM interactions (corresponding nodes are represented using their Entrez Gene ID). Click here for file

Additional file 10 **Yeast interactome.** Simple interaction file (*.txt) containing the integrated network of the yeast interactome (corresponding nodes are represented using their Entrez Gene ID). Protein-protein interactions (marked with *pp*) are undirected while the rest of edges are directed. Click here for file

Additional file 11 **Node attributes for the yeast interactome.** Tab-separated table file (*.txt) file containing various ID(s) for each node in the yeast interactome. Click here for file

Additional file 12 **Code/dataset bundle.** Compressed ZIP file (*.zip) containing all codes and datasets used in this experiment. Click here for file
