# Supplementary material for: Inferring the effective TOR-dependent network: a computational study in yeast
Source: BMC Syst Biol. 2013 Aug 30;7:84. doi: 10.1186/1752-0509-7-84 (PMC4016608; doi:10.1186/1752-0509-7-84)
Supplement: Additional file 12 — Code/dataset bundle. Compressed ZIP file (*.zip) containing all codes and datasets used in this experiment. [file 1752-0509-7-84-S12.zip › experiment/methods/matlab_bgl/doc/html/new_in_3/new_in_3_0.html]

New features in MatlabBGL version 3.0


 


# New features in MatlabBGL version 3.0

Although MatlabBGL 3.0 was never officially released, here are some of it's key features.

## Contents

- Better performance
- Graph construction functions
- Targeted search
- Edge weights
- Matching algorithms
- New graph statistics
- Max-flow algorithms
- Dominator tree
- New utility functions

## Better performance

We redid the backend interface to the BGL routines. This optimization gave a considerable performance increase.

test\_benchmark on MatlabBGL 2.1

```
2008-10-07, Version 2.1, Matlab 2007b, boost 1.33.0,
  g++-3.4 (lib), gcc-? (mex)
```

```
       airfoil       west    cs-stan    minneso      tapir
large   0.223 s    0.024 s    0.390 s    0.073 s    0.046 s
  med     NaN s    0.955 s      NaN s      NaN s    6.621 s
small     NaN s    0.758 s      NaN s      NaN s      NaN s
```

test\_benchmark on MatlabBGL 3.0

```
2008-10-07: Version 3.0, Matlab 2007b, boost 1.34.1,
  g++-4.0 (lib), gcc-? (mex)
```

```
       airfoil       west    cs-stan    minneso      tapir
large   0.183 s    0.017 s    0.222 s    0.048 s    0.037 s
  med     NaN s    0.593 s      NaN s      NaN s    3.901 s
small     NaN s    0.543 s      NaN s      NaN s      NaN s
```

---

## Graph construction functions

MatlabBGL 2.1 had a few graph construction functions. MatlabBGL 3.0 adds the grid\_graph function for line, grid, cube, and
hyper-cube graphs

```
[G xy] = grid_graph(6,5); gplot(G,xy,'.-');
```

In more dimensions...

```
[G xyz] = grid_graph(6,5,3);
G = grid_graph(2,2,2,2);
G = grid_graph([3,3,3,3,3]);
```

---

## Targeted search

The graph search algorithms now let you specify a target vertex that will stop the search early if possible.

```
A = grid_graph(50,50);
tic; d = bfs(A,1,struct()); toc
tic; d = bfs(A,1,struct('target',2)); toc
```

```
Elapsed time is 0.001523 seconds.
Elapsed time is 0.000704 seconds.
```

Also implemented for astar\_search, shortest\_paths, and dfs.

---

## Edge weights

In Matlab, there is no way to create a sparse matrix with a structural non-zero (used for MatlabBGL edges) and a value of
0 (used for MatlabBGL weights). Consequently, it's impossible to run algorithms on graphs where the edge weights are 0.

Consequently, some algorithms now take an 'edge\_weight' parameter that allows you to provide a different set of edge weights
which allow structural non-zeros and 0 values.

This behavior is a bit complicated, so see the REWEIGHTED\_GRAPHS example for more information.

---

## Matching algorithms

While maximum cardinality bipartite matching is just a call to max-flow, general graph matching algorithms are not. MatlabBGL
3.0 contains the matching algorithms in Boost 1.34.0.

```
load('../graphs/matching_example.mat');
m = matching(A);
sum(m>0)/2 % matching cardinality should be 8
```

```
ans =

     8
```

---

## New graph statistics

We added a few new statistics functions.

Test for a topological ordering of a graph (only applies to DAGs or directed acyclic graphs)

```
n = 10; A = sparse(1:n-1, 2:n, 1, n, n); % construct a simple dag
p = topological_order(A);

test_dag(A)
test_dag(cycle_graph(6)) % a cycle is not acyclic!
```

```
ans =

     1


ans =

     0
```

Core numbers can help identify important regions in a graph. MatlabBGL includes weighted and directed core numbers. Also,
the algorithms return the removal time of a particular vertex, which gives interesting graph orderings.

```
% See EXAMPLES/CORE_NUMBERS_EXAMPLE
```

New algorithms for clustering\_coefficients on weighted and directed graphs.

```
A = clique_graph(6) - cycle_graph(6); % A is a clique - a directed cycle
ccfs = clustering_coefficients(A)
B = sprand(A);
ccfs = clustering_coefficients(B)
C = A|A'; % now it's a full clique again
ccfs = clustering_coefficients(C)
```

```
ccfs =

    0.7600
    0.7600
    0.7600
    0.7600
    0.7600
    0.7600


ccfs =

    0.4543
    0.4064
    0.4363
    0.4310
    0.4109
    0.4180


ccfs =

     1
     1
     1
     1
     1
     1
```

---

## Max-flow algorithms

Since Boost added the Kolmogorov max-flow function, we added the full collection of flow algorithms to MatlabBGL.

```
load('../graphs/max_flow_example.mat');

push_relabel_max_flow(A,1,8)
kolmogorov_max_flow(A,1,8)
edmunds_karp_max_flow(A,1,8)

max_flow(A,1,8,struct('algname','push_relabel'));
max_flow(A,1,8,struct('algname','kolmogorov'));
max_flow(A,1,8,struct('algname','edmunds_karp'));
```

```
ans =

     4


ans =

     4


ans =

     4
```

---

## Dominator tree

Dominator trees are relations about presidence in certain types of graphs. These are also called flow-graphs.

```
load('../graphs/dominator_tree_example.mat');
p = lengauer_tarjan_dominator_tree(A,1);
```

---

## New utility functions

MatlabBGL 3.0 introduces some new utility functions.

The output of a shortest path algorithm is a predecessor matrix. To convert these predecessor relationships to a path, use
the path\_from\_pred function.

```
[A xy] = grid_graph(6,5); n= size(A,1);
[d dt pred] = bfs(A,1); %
path = path_from_pred(pred,n) % sequence of vertices to upper corner
```

```
path =

     1     2     3     4     5     6    12    18    24    30
```

Let's draw the path

```
gplot(A,xy,'r.-');
[px,py]=gplot(sparse(path(1:end-1),path(2:end),1,n,n),xy,'-');
hold on; plot(px,py,'-','LineWidth',2); hold off;
```

We can also create a full shortest path tree using the tree\_from\_pred function.

```
T = tree_from_pred(pred);
gplot(A,xy,'r.-');
[px,py]=gplot(T,xy,'-');
hold on; plot(px,py,'-','LineWidth',2); hold off;
```

Finally, there are a few new routines to make working with reweighted graphs easier. See EXAMPLES/REWEIGHTED\_GRAPHS for information
about the INDEXED\_SPARSE and EDGE\_WEIGHT\_INDEX functions.

---
