# Supplementary material for: Inferring the effective TOR-dependent network: a computational study in yeast
Source: BMC Syst Biol. 2013 Aug 30;7:84. doi: 10.1186/1752-0509-7-84 (PMC4016608; doi:10.1186/1752-0509-7-84)
Supplement: Additional file 12 — Code/dataset bundle. Compressed ZIP file (*.zip) containing all codes and datasets used in this experiment. [file 1752-0509-7-84-S12.zip › experiment/methods/matlab_bgl/doc/html/new_in_4/new_in_4_0.html]

New features in MatlabBGL version 4.0


 


# New features in MatlabBGL version 4.0

MatlabBGL 3.0 was only released as a beta test. Version 4.0 is a full release with quite a few new features.

## Contents

- Improved reweighted graphs
- Graph layout algorithms
- Planar graph algorithms
- New option syntax

## Improved reweighted graphs

In MatlabBGL 3.0, reweighted graphs were a pain to use. Now they are simple! We combine a structural matrix with a weight
matrix. As(i,j)=1 if there is an edge between vertex i and j and A(i,j)=wij where wij is the weight of the edge between i
and j.

```
As = cycle_graph(6,'directed',0); % compute a 6 node cycle graph
A = As; % set all the weights to be one initially
A(2,3) = 0; A(3,2) = 0; % make one edge have zero weight
fprintf('Edges\n');
full(As)
fprintf('Weights\n');
full(A)
```

```
Edges

ans =

     0     1     0     0     0     1
     1     0     1     0     0     0
     0     1     0     1     0     0
     0     0     1     0     1     0
     0     0     0     1     0     1
     1     0     0     0     1     0

Weights

ans =

     0     1     0     0     0     1
     1     0     0     0     0     0
     0     0     0     1     0     0
     0     0     1     0     1     0
     0     0     0     1     0     1
     1     0     0     0     1     0
```

Note that As is given as the graph in the following call, not A!

```
[d pred] = shortest_paths(As,1,'edge_weight',edge_weight_vector(As,A));
d(3) % distance from vertex 1 to vertex 3 should be just 1!
```

```
ans =

     1
```

---

## Graph layout algorithms

Sometimes, it's really nice to see a picture of your graph. The BGL implements a few graph layout algorithms and so these
are now in MatlabBGL 4.0!

```
G = grid_graph(6,5);
X = kamada_kawai_spring_layout(G);
gplot(G,X,'.-');
```

```
G = grid_graph(6,5);
X = fruchterman_reingold_force_directed_layout(G);
gplot(G,X,'.-');
```

```
G = grid_graph(6,5);
X = gursoy_atun_layout(G);
gplot(G,X,'.-');
```

 

---

## Planar graph algorithms

The Boost Graph Library received a new suite of planar graph algorithms. These are now in MatlabBGL too.

A grid in the xy plane is a planar graph.

```
G = grid_graph(6,5);
is_planar = boyer_myrvold_planarity_test(G)
```

```
is_planar =

     1
```

Recall that K\_5 (the clique on 5 vertices) is not a planar graph. Let's see what happens.

```
K5 = clique_graph(5);
is_planar = test_planar_graph(K5) % helpful wrapper
```

```
is_planar =

     0
```

We can also draw planar graphs

```
G = grid_graph(6,5);
X = chrobak_payne_straight_line_drawing(G);
gplot(G,X,'.-'); % it looks a little different!
```

 

---

## New option syntax

You probably noticed that the "struct" command that permeated MatlabBGL calls before is gone in these examples. We've moved
to a new option syntax that gives you the choice between the MatlabBGL struct style arguments and a list of key-value pairs

We'll look at spanning trees on the clique graph with 5 vertices. Using Prim's algorithm, the spanning tree we get depends
on the root. We always get a star graph rooted at the vertex we pick as the root.

```
G = clique_graph(5);
```

Old style

```
full(mst(G,struct('root',5,'algname','prim')))
```

```
ans =

     0     0     0     0     1
     0     0     0     0     1
     0     0     0     0     1
     0     0     0     0     1
     1     1     1     1     0
```

New style

Just to make sure it works

```
full(mst(G,'root',1,'algname','prim'))
```

```
ans =

     0     1     1     1     1
     1     0     0     0     0
     1     0     0     0     0
     1     0     0     0     0
     1     0     0     0     0
```

---
