# Supplementary material for: Inferring the effective TOR-dependent network: a computational study in yeast
Source: BMC Syst Biol. 2013 Aug 30;7:84. doi: 10.1186/1752-0509-7-84 (PMC4016608; doi:10.1186/1752-0509-7-84)
Supplement: Additional file 12 — Code/dataset bundle. Compressed ZIP file (*.zip) containing all codes and datasets used in this experiment. [file 1752-0509-7-84-S12.zip › experiment/methods/matlab_bgl/doc/html/red_black/red_black.html]

Red-Black Ordering with MatlabBGL


 


# Red-Black Ordering with MatlabBGL

In this example, we will use the MatlabBGL library to compute the red-black ordering of a matrix. For certain matrices, the
red-black ordering does not exist, but if a red-black ordering exists, then this algorithm will find it.

A matrix has a red-black ordering if the directed graph of the matrix elements is bipartite. To find a bipartite ordering
of the matrix, we will use the MatlabBGL bfs command.

## Contents

- Generating a Matrix
- Finding the Red-Black ordering

## Generating a Matrix

If you already have a matrix you want to use, you can skip this step. First, we generate a second order finite difference
approximation to the Laplacian operator on a rectangular domain. This matrix does have a red-black ordering.

n is the number of points used to discretize each dimension. N is the total number rows and columns in the matrix/graph.

```
n = 55;
N = n*n;
```

This set of commands creates a pentadiagonal matrix.

```
A = delsq(numgrid('S',n+2));
```

Now we visualize the matrix.

```
spy(A)
```

The matrix A we just created is block tridiagonal, although this is (perhaps) not obvious from the plot.

We know, analytically, that this matrix has a red black ordering given by the odd points and then the even points, when indexed
in the current order.

```
p = [1:2:N 2:2:N];
spy(A(p,p));
```

Usually, it's a little easier to see a red-black ordering using the following command.

```
spy(A(p,p) - diag(diag(A(p,p))));
hold on;
plot([size(A,2)/2 size(A,2)/2],[0 size(A,1)], 'k-');
plot([0 size(A,2)],[size(A,1)/2 size(A,1)/2], 'k-');
hold off;
```

Now we can see that a matrix with a red-black ordering only has non-zero dots in the upper right and lower left boxes.

---

## Finding the Red-Black ordering

To find the red-black ordering for an arbitrary matrix (if we do not know it analytically) is easy using MatlabBGL.

The key idea is to realize that a red-black ordering is equivalent with the partition of vertices in a bipartite graph. Once
we see the problem in this light, we can quickly come up with an algorithm that yields a potential red-black ordering.

We begin by picking an arbitrary vertex and look at how far a breadth first search goes at every step. To find the bipartition,
we look at all vertices which are an even distance from the root and all the vertices which are an odd distance from the root.
If the matrix has a red-black ordering or is a bipartite graph, this algorithm will find it.

Implementing this algorithm is trivial using the MatlabBGL library. First, we compute a breadth first search on the graph
and store the distance each vertex is from the root. Because we really do not care, we'll choose vertex 1 (row 1) of the
matrix as the root vertex.

```
d = bfs(A,1);
```

Now we find the even and odd partitions

```
d_even = find(mod(d,2) == 0);
d_odd = find(mod(d,2) == 1);
```

Getting the actual permutation of the matrix is simple, we list the odd vertices and then the even vertices

```
p = [d_odd' d_even'];
```

Computing the same plot shows we found the red-black ordering!

```
spy(A(p,p) - diag(diag(A(p,p))));
hold on;
plot([size(A,2)/2 size(A,2)/2],[0 size(A,1)], 'k-');
plot([0 size(A,2)],[size(A,1)/2 size(A,1)/2], 'k-');
hold off;
```

and that's it! MatlabBGL and the bfs command made this problem simple!

---
