# Supplementary material for: Inferring the effective TOR-dependent network: a computational study in yeast
Source: BMC Syst Biol. 2013 Aug 30;7:84. doi: 10.1186/1752-0509-7-84 (PMC4016608; doi:10.1186/1752-0509-7-84)
Supplement: Additional file 12 — Code/dataset bundle. Compressed ZIP file (*.zip) containing all codes and datasets used in this experiment. [file 1752-0509-7-84-S12.zip › experiment/methods/matlab_bgl/doc/html/record_alg/record_alg.html]

Recording algorithm behavior with MatlabBGL


 


# Recording algorithm behavior with MatlabBGL

In this example, we will write a simple visitor that outputs an algorithm's behavior. The algorithm we will examine is dijkstra\_sp.
To examine the runtime behavior we will use a visitor which outputs a string every time a function is called.

## Contents

- Setup
- Calling dijkstra\_sp
- Understanding the output

## Setup

To begin, we load a graph.

```
load ../graphs/clr-25-2.mat
```

Next, let's check the documentation to see which functions to implement for the visitor

```
help dijkstra_sp
```

```
  DIJKSTRA_SP Compute the weighted single source shortest path problem.
 
  Dijkstra's algorithm for the single source shortest path problem only
  works on graphs without negative edge weights.
 
  This method works on weighted directed graphs without negative edge
  weights.
  The runtime is O(V log (V)).
 
  See the shortest_paths function for calling information.  This function 
  just calls shortest_paths(...,struct('algname','dijkstra'));
 
  The options structure can contain a visitor for the Dijkstra algorithm.  
 
  See http://www.boost.org/libs/graph/doc/DijkstraVisitor.html for a 
  description of the events.
  
  visitor is a struct with the following optional fields
     vis.initialize_vertex(u)
     vis.discover_vertex(u)
     vis.examine_vertex(u)
     vis.examine_edge(ei,u,v)
     vis.edge_relaxed(ei,u,v)
     vis.edge_not_relaxed(ei,u,v)
     vis.finish_vertex(u)
  Each visitor parameter should be a function pointer, which returns 0
  if the shortest path search should stop.  (If the function does not 
  return anything, the algorithm continues.)
 
  Example:
     load graphs/clr-25-2.mat
     dijkstra_sp(A,1)
 
  See also SHORTEST_PATHS, BELLMAN_FORD_SP.
```

The help states that dijkstra\_sp allows visitors functions for initialize\_vertex, discover\_vertex, examine\_vertex, examine\_edge,
edge\_relaxed, edge\_not\_relaxed, and finish\_vertex.

Rather than implementing 7 functions ourselves, we define two helper functions. These helper functions return functions themselves.
There is one helper that returns a vertex visitor function and one helper than returns an edge visitor function.

```
vertex_vis_print_func = @(str) @(u) ...
    fprintf('%s called on %s\n', str, char(labels{u}));
edge_vis_print_func = @(str) @(ei,u,v) ...
    fprintf('%s called on (%s,%s)\n', str, char(labels{u}), char(labels{v}));
```

These anonymous functions return functions themselves.

```
ev_func = vertex_vis_print_func('examine_vertex');
ev_func(1)
```

```
examine_vertex called on s
```

I hope you see how these functions are useful in saving quite a bit of typing.

---

## Calling dijkstra\_sp

We are almost done. Now, we just have to setup the visitor structure to pass to the dijkstra\_sp call.

```
vis = struct();
vis.initialize_vertex = vertex_vis_print_func('initialize_vertex');
vis.discover_vertex = vertex_vis_print_func('discover_vertex');
vis.examine_vertex = vertex_vis_print_func('examine_vertex');
vis.finish_vertex = vertex_vis_print_func('finish_vertex');
vis.examine_edge = edge_vis_print_func('examine_edge');
vis.edge_relaxed = edge_vis_print_func('edge_relaxed');
vis.edge_not_relaxed = edge_vis_print_func('edge_not_relaxed');
```

With the visitor setup, there is hardly any work left.

```
dijkstra_sp(A,1,struct('visitor', vis));
```

```
initialize_vertex called on s
initialize_vertex called on u
initialize_vertex called on x
initialize_vertex called on v
initialize_vertex called on y
discover_vertex called on s
examine_vertex called on s
examine_edge called on (s,u)
edge_relaxed called on (s,u)
discover_vertex called on u
examine_edge called on (s,x)
edge_relaxed called on (s,x)
discover_vertex called on x
finish_vertex called on s
examine_vertex called on u
examine_edge called on (u,x)
edge_not_relaxed called on (u,x)
examine_edge called on (u,v)
edge_relaxed called on (u,v)
discover_vertex called on v
finish_vertex called on u
examine_vertex called on x
examine_edge called on (x,u)
examine_edge called on (x,v)
edge_not_relaxed called on (x,v)
examine_edge called on (x,y)
edge_relaxed called on (x,y)
discover_vertex called on y
finish_vertex called on x
examine_vertex called on v
examine_edge called on (v,y)
edge_not_relaxed called on (v,y)
finish_vertex called on v
examine_vertex called on y
examine_edge called on (y,s)
examine_edge called on (y,v)
finish_vertex called on y
```

---

## Understanding the output

To understand the output, we find it helpful to have a copy of Introduction to Algorithms by Cormen, Leiserson, and Rivest.
The source for the graph is Figure 25-2 in that book and the authors use the graph to illustrate how Dijkstra's algorithm
runs. In particular, Figure 25-5 shows a sample run of Dijkstra's algorithm.

Perhaps the first thing to notice is that the initialize vertex visitor is never called. This results from an error in the
MatlabBGL and Boost documentation. Once it is resolved, we will update the MatlabBGL documentation to match the Boost graph
library.

The results: discover\_vertex is called before examine\_vertex. For the edges, examine\_edge is always called before either
edge\_relaxed or edge\_not\_relaxed. The edges that are relaxed are the shaded edges in Figure 25-5.

Finally, finish vertex is called on a vertex after all of its edges have been examined and possibly relaxed.

---
