# Supplementary material for: Inferring the effective TOR-dependent network: a computational study in yeast
Source: BMC Syst Biol. 2013 Aug 30;7:84. doi: 10.1186/1752-0509-7-84 (PMC4016608; doi:10.1186/1752-0509-7-84)
Supplement: Additional file 12 — Code/dataset bundle. Compressed ZIP file (*.zip) containing all codes and datasets used in this experiment. [file 1752-0509-7-84-S12.zip › experiment/methods/matlab_bgl/doc/html/planar_graphs/planar_graphs.html]

Planar graphs in MatlabBGL


 


# Planar graphs in MatlabBGL

In version 1.35.0, the Boost Graph Library added a large suite of planar graph algorithms.

## Contents

- Planarity testing
- A (planar?) road network
- Planar embeddings
- Conclusion

## Planarity testing

Two functions help test if a graph is planar. The algorithm is the Boyer-Myrvold planarity tester.

```
K5 = clique_graph(5);
test_planar_graph(K5)
```

```
ans =

     0
```

Of course K\_5 isn't a planar graph. To get more information about why it isn't planar, we use the boyer\_myrvold\_planarity\_test
function. When a graph isn't planar, this function will isolate a Kuratowski subgraph.

```
[is_planar K] = boyer_myrvold_planarity_test(K5);
is_planar
full(K)
```

```
is_planar =

     0


ans =

     0     1     1     1     1
     1     0     1     1     1
     1     1     0     1     1
     1     1     1     0     1
     1     1     1     1     0
```

A Kuratowski subgraph is a certificate that a graph isn't planar. A Kuratowski subgraph must contract to either K\_5 or K\_3,3
(a bipartite clique). In this case, the graph was K\_5, and so K was the entire graph.

---

## A (planar?) road network

Let's have some fun! Let's look at a road network.

```
load('../graphs/minnesota.mat');
gplot(A,xy,'.-');
```

```
test_planar_graph(A)
```

```
ans =

     0
```

What? The road network isn't planar? Let's see what is going on here.

```
[is_planar K] = boyer_myrvold_planarity_test(A);

gplot(K,xy,'.-');
```

It looks like there are a lot of tree-like portions. Those shouldn't be the problem, let's remove them.

```
cn = core_numbers(K);
K2 = K;
K2(cn<2,cn<2) = 0;
gplot(K2,xy,'.-');
```

We'd better check the graph is still Kuratowski. There's a function called is\_kuratowski\_graph that does just this task.

```
is_kuratowski_graph(K2)
```

```
ans =

     1
```

Well, that looks more helpful, but I don't see the planarity problem. Now, let's try contracting edges. What the following
code does is to look for vertices of degree 2 (pieces of a line) and remove the intermediate vertex. In Matlab it isn't very
efficent code, but this graph only has a few edges (~1000) at this point, so it'll be fast enough.

```
Kcur = K2;

rand('state',0); % reset for deterministic results
for ntimes=1:20
    % compute the degree of all edges
    d = sum(Kcur,2);
    % pick an independent set of vertices with degree 2
    s = d==2;
    s = s.*round(rand(size(s))); % randomly pick entries
    a = Kcur*s; % follow one edge
    s = s&~a; % remove dependent edges
    a = Kcur*double(s);
    fprintf('check for is: %i\n', full(sum(a&s))==0); % verify indep set.

    % contract the edges
    for k=find(s)'
        ns = find(Kcur(:,k));
        Kcur(ns(1),ns(2)) = 1;
        Kcur(:,k) = 0;
        Kcur(k,:) = 0;
    end
    Kcur = Kcur|Kcur';
end

% plot the graph after contraction in red
gplot(K2,xy,'.-'); hold on; gplot(Kcur,xy,'r.-'); hold off;
```

```
check for is: 1
check for is: 1
check for is: 1
check for is: 1
check for is: 1
check for is: 1
check for is: 1
check for is: 1
check for is: 1
check for is: 1
check for is: 1
check for is: 1
check for is: 1
check for is: 1
check for is: 1
check for is: 1
check for is: 1
check for is: 1
check for is: 1
check for is: 1
```

Ahah, now we see the problem. (Try to untangle the red graph!)

Now that we see the problem, I think it's clear what we should have done from the beginning...

```
d = sum(K);
max(d)
```

```
ans =

   (1,1)        3
```

The maximum degree is 3, so the subgraph must be isomorphic to K\_3,3.

```
d3= d==3;
sum(d3);
```

That is the problem with the graph, but why doesn't the display show it? Well, it does.

```
gplot(K2,xy,'.-'); hold on; gplot(Kcur,xy,'r.-'); hold off;
xlim([-95.2092  -94.5842]);
ylim([   43.5903   43.7778]);
```

It looks like there is a vertex of degree 4 in the middle. Unfortunately, that is just 2 paths crossing. Zooming in further,
there are actually two vertices there! That's the problem!

And so, here is a problem for you:

Problem, automatically identify the following pairs of vertices as problematic for the planar embedding [2546,2547] [1971,1975]
[1663,1666] Find another pair that prevents a planar embedding of the graph.

---

## Planar embeddings

To investigate planar embeddings, let's start with the road network again.

```
load('../graphs/minnesota.mat');
test_planar_graph(A(1:500,1:500))
```

```
ans =

     1
```

Good, we found a planar region!

```
A = A(1:500,1:500);
xy = xy(1:500,:);

gplot(A,xy,'.-');
```

Let's compute it's planar embedding

```
X = chrobak_payne_straight_line_drawing(A);
gplot(A,X,'.-');
```

Well, that isn't quite as helpful, but now you know how to compute a straight line drawing. The straight line drawing is
computed from a maximal planar graph. A maximal planar graph cannot have any additional edges and still be planar.

```
M = make_maximal_planar(A);
gplot(M,X,'.-');
```

 

---

## Conclusion

That's it for our brief tour of planar graph algorithms in MatlabBGL. See the BGL documentation pages on planar graph algorithms
for more information.

Planar Graphs in the Boost Graph Library

---
