# Supplementary material for: Inferring the effective TOR-dependent network: a computational study in yeast
Source: BMC Syst Biol. 2013 Aug 30;7:84. doi: 10.1186/1752-0509-7-84 (PMC4016608; doi:10.1186/1752-0509-7-84)
Supplement: Additional file 12 — Code/dataset bundle. Compressed ZIP file (*.zip) containing all codes and datasets used in this experiment. [file 1752-0509-7-84-S12.zip › experiment/methods/matlab_bgl/doc/html/reweighted_graphs/reweighted_graphs.html]

Reweighted graphs in MatlabBGL


 


# Reweighted graphs in MatlabBGL

Matlab sparse matrices only support non-zero values. Because the structure of the sparse matrix is used to infer the edges
of an underlying graph this limitation prevents MatlabBGL from trivially addressing graphs with 0-weight edges.

To fix this problem, codes that accept a weighted graph allow the user to specify a vector of edge weights for each edge in
the graph using the optional 'weights' parameter. Using the 'weights' parameter correctly can be difficult due to issues
with how edges are indexed in MatlabBGL.

## Contents

- Disclaimer
- "I just want the simpliest solution..."
- A first attempt
- A first solution
- A simplified solution
- An undirected solution
- The undirected simplification
- Summary

## Disclaimer

The details of this section are complicated. This means their implementation is error-prone. If you get strange behavior,
please let me know.

---

## "I just want the simpliest solution..."

**new in version 4.0** In this section, we'll see the really-easy but somewhat expensive way of reweighting a graph. I'll run through all the cases
detailed below with the simple code. If you just need something to work and don't necessarily need to know about all the
details, this section is for you!

Let's compute shortest paths in a cycle graph with only one weighted edge. The simple case requires a structural and weight
matrix.

n will be the total size of the graph, and u and v will be the special vertices connected with a weight one edge.

```
n = 8; % it's just an example, so let's make it small.
u = 1;
v = 2;
```

These commands create an undirected cycle graph. The cycle is ... n - 1 - 2 - ... - n-1 - n - 1 ... where the weight on every edge is 0 except for the edge between vertex u,v. Notice that the edge list is already symmetric.

This setup means that while there is a weight 1 edge between u and v, the shortest path, or smallest weight path, is actually
the path from u, circling every vertex except v and so d(v) should be 0.

```
E = [1:n 2:n 1; 2:n 1 1:n]';
w = [1 zeros(1,n-1) 1 zeros(1,n-1)]';

A = sparse(E(:,1), E(:,2), w, n, n); % create a weighted sparse matrix
As = sparse(E(:,1), E(:,2), true, n, n); % create a structural sparse matrix
```

The relationship between As and A is that As should have a non-zero value for every edge, but the values of As will be ignored and the computation will proceed with the values in the corresponding spots in the matrix A.

The wrong way to compute shortest paths.

```
[d pred] = shortest_paths(A,u);
d(v)
```

```
ans =

     1
```

The right way to compute shortest paths.

```
[d pred] = shortest_paths(As,u,struct('edge_weight',edge_weight_vector(As,A)));
d(v)
```

```
ans =

     0
```

That's better, d(v) = 0 as expected.

---

## A first attempt

- Correct for version 3.0 \* A trivial example graph to illustrate the problem that occurs with 0 weighted graphs occurs even
  with a simple cycle. Suppose that the graph corresponding to adjacency matrix A is a symmetric cycle where all edges have
  weight 0 except for one edge between vertices (1,2).

These commands create an undirected cycle graph. The cycle is ... n - 1 - 2 - ... - n-1 - n - 1 ... where the weight on every edge is 0 except for the edge between vertex u,v. Notice that the edge list is already symmetric.

```
E = [1:n 2:n 1; 2:n 1 1:n]';
w = [1 zeros(1,n-1) 1 zeros(1,n-1)]';

A = sparse(E(:,1), E(:,2), w, n, n);
```

The shortest weighted path between u and v is then through the vertex n because traversing the cycle in the other direction
will use the u,v edge of weight 1. Let's check this with the shortest\_paths function.

```
[d pred] = shortest_paths(A,u);
d(v)
```

```
ans =

     1
```

That is weird, there is a u-v path of length 0 in the graph! Let's see what path the shortest path algorithm chose.

```
path_from_pred(pred,v)
```

```
ans =

     1     2
```

The path it chose was from u to v directly, taking the weight 1 edge. Let's look at the sparse matrix.

```
A
```

```
A =

   (2,1)        1
   (1,2)        1
```

There are only two edges in the matrix corresponding to our symmetric weight 1 edge between u and v. This happens because
Matlab removes all 0 weight edges from the graph.

---

## A first solution

The solution to the problem is to use the 'edge\_weight' optional parameter to the shortest\_paths function to give it a set
of weights to use for each edge.

```
help shortest_paths
```

```
  SHORTEST_PATHS Compute the weighted single source shortest path problem.
 
  [d pred] = shortest_paths(A,u) returns the distance (d) and the predecessor
  (pred) for each of the vertices along the shortest path from u to every
  other vertex in the graph.  
  
  ... = shortest_paths(A,u,...) takes a set of
  key-value pairs or an options structure.  See set_matlab_bgl_options
  for the standard options. 
    options.algname: the algorithm to use 
        [{'auto'} | 'dijkstra' | 'bellman_ford' | 'dag']
    options.inf: the value to use for unreachable vertices 
        [double > 0 | {Inf}]
    options.target: a special vertex that will stop the search when hit
        [{'none'} | any vertex number besides the u]; target is ignored if
        visitor is set.
    options.visitor: a structure with visitor callbacks.  This option only
        applies to dijkstra or bellman_ford algorithms.  See dijkstra_sp or
        bellman_ford_sp for details on the visitors.
    options.edge_weight: a double array over the edges with an edge
        weight for each edge, see EDGE_INDEX and EXAMPLES/REWEIGHTED_GRAPHS
        for information on how to use this option correctly
        [{'matrix'} | length(nnz(A)) double vector]
 
  Note: if you need to compute shortest paths with 0 weight edges, you must
  use an edge_weight vector, see the examples for details.
 
  Note: 'auto' cannot be used with 'nocheck' = 1.  The 'auto' algorithm
  checks if the graph has negative edges and uses bellman_ford in that
  case, otherwise, it uses 'dijkstra'.  In the future, it may check if the
  graph is a dag and use 'dag'.  
 
  Example:
     load graphs/clr-25-2.mat
     shortest_paths(A,1)
     shortest_paths(A,1,struct('algname','bellman_ford'))
 
  See also DIJKSTRA_SP, BELLMAN_FORD_SP, DAG_SP
```

Well, shortest\_paths says to read this document, so you are on the right track! It also has a pointer to the function edge\_weight\_index.
Let's look at that function

```
help edge_weight_index
```

```
  EDGE_WEIGHT_INDEX Build a conformal matrix of edge index values for a graph.
 
  [eil Ei] = edge_weight_index(As) returns a vector where 
    As(i,j) not= 0 implies Ei(i,j) not= 0 and Ei(i,j) = eil(i)
  for an integer value of eil(i) that corresponds to the edge index value
  passed in the visitors.  
 
  The input matrix A should be a structural matrix with a non-zero value
  for each edge.  The matrix Ei gives an index for each edge in the graph,
  and the vector eil will reorder a vector of edge weights to an appropriate
  input for 'edge_weight' parameter of a function call.
 
  The edge_weight_index function assists writing codes that use the
  edge_weight parameter to reweight a graph based on a vector of weights
  for the graph or using the ei parameter from an edge visitor.  It is 
  critical to obtain high performance when
 
  i) constructing algorithms that use 0 weighted edges
  ii) constructing algorithms that change edge weights often.
 
  See the examples reweighted_edges and edge_index_visitor for more
  information.
 
  ... = edge_weight_index(A,...) takes a set of
  key-value pairs or an options structure.  See set_matlab_bgl_options
  for the standard options. 
     options.undirected: output edge indices for an undirected graph [{0} | 1]
         see Note 1.
 
  Note 1: For an undirected graph, the edge indices of the edge corresponding
  to (u,v) and (v,u) are the same.  Consequently, Ei is a symmetric matrix,
  using this option allows only one value for an undirected edge.
 
  Example:
    load('graphs/bfs_example.mat');
    [eil Ei] = edge_weight_index(A,struct('undirected',1));
    edge_rand = rand(num_edges(A)/2,1);
    [iu ju] = find(triu(A,0));
    Av = sparse(iu,ju,edge_rand,size(A,1),size(A,1)); Av = Av + Av';
    ee = @(ei,u,v) fprintf('examine_edge %2i, %1i, %1i, %4f, %4f, %4f\n', ...
                ei, u, v, edge_rand(eil(ei)), Av(u,v), edge_rand(Ei(u,v)));
    breadth_first_search(A,1,struct('examine_edge', ee));
 
  See also INDEXED_SPARSE
```

This function claims to help us. It requires building a structural matrix which has a non-zero for each edge in the graph.
Let's do that.

```
As = sparse(E(:,1), E(:,2), 1, n, n)
```

```
As =

   (2,1)        1
   (8,1)        1
   (1,2)        1
   (3,2)        1
   (2,3)        1
   (4,3)        1
   (3,4)        1
   (5,4)        1
   (4,5)        1
   (6,5)        1
   (5,6)        1
   (7,6)        1
   (6,7)        1
   (8,7)        1
   (1,8)        1
   (7,8)        1
```

Now the matrix has all of the required edges. According to the edge\_weight\_index function, it returns both a matrix and an
index vector. The index vector is a way to permute an intelligently ordered set of edge weights to the order that MatlabBGL
requires the edge weights.

```
[ei Ei] = edge_weight_index(As);

full(Ei)
ei
```

```
ans =

     0     3     0     0     0     0     0    15
     1     0     5     0     0     0     0     0
     0     4     0     7     0     0     0     0
     0     0     6     0     9     0     0     0
     0     0     0     8     0    11     0     0
     0     0     0     0    10     0    13     0
     0     0     0     0     0    12     0    16
     2     0     0     0     0     0    14     0


ei =

     3
    15
     1
     5
     4
     7
     6
     9
     8
    11
    10
    13
    12
    16
     2
    14
```

Now let's create a new edge weight vector for this graph corresponding to all the edges we want. Each non-zero in the matrix
should have an associated edge weight. Most the edge weights in this case are 0, so it makes it simple.

```
ew = zeros(nnz(As),1);
ew(Ei(u,v)) = 1;
ew(Ei(v,u)) = 1;

[d pred] = shortest_paths(As,u,struct('edge_weight', ew(ei)));

path_from_pred(pred,v)
```

```
ans =

     1     8     7     6     5     4     3     2
```

Excellent, now the shorest path avoids the edge (u,v) as we would expect it.

---

## A simplified solution

The current example is somewhat tedious because we have to create the sparse matrix, then create the edge index matrix, and
finally create and edit the edge weight array.

The indexed\_sparse function makes the process easier.

Recall that using the sparse function directly generated an incorrect graph adjacency matrix.

```
A = sparse(E(:,1), E(:,2), w, n, n)
```

```
A =

   (2,1)        1
   (1,2)        1
```

The indexed\_sparse function is designed as a replacement for sparse where the adjacency matrix must be indexed using the edge\_weight\_index
or contains 0 weight edges.

```
help indexed_sparse
```

```
  INDEXED_SPARSE Create a sparse matrix with indexed edges.
 
  [As,A,eil,Ei] = indexed_sparse(i,j,v,m,n) creates a sparse matrix A just
  like A = sparse(i,j,v,m,n).  However, indexed_sparse returns additional
  information.  The matrix As is a structural matrix for A which
  corresponds to As = sparse(i,j,1,m,n).  Thus, As(i,j) != 0 for all edges.
  The vector eil is a permutation for the vector v, such that v(eil) is the
  correct input for the edge_weight parameter.  The matrix Ei lists the
  index of each edge in the vector v, so that 
  A = sparse(j,i,v(nonzeros(Ei)),m,n)' unless options.istrans = 0.  
 
  This function handles the case when v(k) == 0.  For v(k) = 0,
  A(i(k),j(k)) = 0, but As(i(k),j(k)) = 1, and the vector v(eil) provides
  an appropriate input to the edge_weight parameter for all the algorithms.
   
  See the examples reweighted_edges for more information.
 
  ... = indexed_sparse(A,...) takes a set of
  key-value pairs or an options structure.  See set_matlab_bgl_options
  for the standard options. 
     options.undirected: output edge indices for an undirected graph [{0} | 1]
       See the note about undirected inputs.
 
  Note (Undirected inputs): If options.undirected = 1, the input to the
  graph still must contain both undirected edges and the corresponding
  weight.  
 
  Example:
    % see example/reweighted_edges
```

From the documentation of indexed\_sparse, the first two return values are the structural sparse matrix (As) and the sparse
matrix (A) that sparse would have returned. The final two return values are the edge index list that edge\_weight\_index returns
as well as the edge index matrix.

```
% save the old Ei as an example
old_Ei = Ei;

[As A eil Ei] = indexed_sparse(E(:,1), E(:,2), w, n, n);

fprintf('old_Ei = \n\n');
disp(full(old_Ei));
fprintf('Ei = \n\n');
disp(full(Ei))
```

```
old_Ei = 

     0     3     0     0     0     0     0    15
     1     0     5     0     0     0     0     0
     0     4     0     7     0     0     0     0
     0     0     6     0     9     0     0     0
     0     0     0     8     0    11     0     0
     0     0     0     0    10     0    13     0
     0     0     0     0     0    12     0    16
     2     0     0     0     0     0    14     0

Ei = 

     0     1     0     0     0     0     0    16
     9     0     2     0     0     0     0     0
     0    10     0     3     0     0     0     0
     0     0    11     0     4     0     0     0
     0     0     0    12     0     5     0     0
     0     0     0     0    13     0     6     0
     0     0     0     0     0    14     0     7
     8     0     0     0     0     0    15     0
```

Note that the edge indices changed between the two calls. The reason for this change is that indexed\_sparse generates edge
indices based on order of E(:,1) and E(:,2). Consequently, this function is much easier to use when you already have a set
of weighted edges.

In this case, we don't have to create the ew array again! (Note that the call uses eil instead of ei.)

```
[d pred] = shortest_paths(As,u,struct('edge_weight', w(eil)));

path_from_pred(pred,v)
```

```
ans =

     1     8     7     6     5     4     3     2
```

---

## An undirected solution

The situation for undirected graphs is more complicated. The trouble with the previous solution is that each directed edge
had its own weight in the vector w. For an undirected graph, we really want each undirected edge to have a single weight,
so the natural length of v would be nnz(A)/2 instead of nnz(A).

However, MatlabBGL really treats all problems as directed graphs, so it will need a vector w of length nnz(A), but that vector
should satisfy the requirement w(ei1) = w(ei2) if the edges corresponding to ei1 and ei2 are (i,j) and (j,i), respectively.

Again, the edge\_weight\_index function provides a solution to this problem. We just have to tell edge\_weight\_index we have
an undirected graph.

Let's start with the same sparse matrix

```
As = sparse(E(:,1), E(:,2), 1, n, n)
```

```
As =

   (2,1)        1
   (8,1)        1
   (1,2)        1
   (3,2)        1
   (2,3)        1
   (4,3)        1
   (3,4)        1
   (5,4)        1
   (4,5)        1
   (6,5)        1
   (5,6)        1
   (7,6)        1
   (6,7)        1
   (8,7)        1
   (1,8)        1
   (7,8)        1
```

Here we use the edge\_weight\_index

```
[ei Ei] = edge_weight_index(As,struct('undirected',1));
full(Ei) % look at the matrix
```

```
ans =

     0     1     0     0     0     0     0     7
     1     0     2     0     0     0     0     0
     0     2     0     3     0     0     0     0
     0     0     3     0     4     0     0     0
     0     0     0     4     0     5     0     0
     0     0     0     0     5     0     6     0
     0     0     0     0     0     6     0     8
     7     0     0     0     0     0     8     0
```

Let's create the edge weight vector

```
ew = zeros(nnz(As)/2,1); % only half as many zeros here.
ew(Ei(u,v)) = 1; % and we only needed to set one entry to 0

[d pred] = shortest_paths(As,u,struct('edge_weight', ew(ei)));

path_from_pred(pred,v)
```

```
ans =

     1     8     7     6     5     4     3     2
```

And we get the same output as before!

---

## The undirected simplification

You can probably guess that the simplification for undirected graphs will use the indexed\_sparse call again too.

```
help indexed_sparse
```

```
  INDEXED_SPARSE Create a sparse matrix with indexed edges.
 
  [As,A,eil,Ei] = indexed_sparse(i,j,v,m,n) creates a sparse matrix A just
  like A = sparse(i,j,v,m,n).  However, indexed_sparse returns additional
  information.  The matrix As is a structural matrix for A which
  corresponds to As = sparse(i,j,1,m,n).  Thus, As(i,j) != 0 for all edges.
  The vector eil is a permutation for the vector v, such that v(eil) is the
  correct input for the edge_weight parameter.  The matrix Ei lists the
  index of each edge in the vector v, so that 
  A = sparse(j,i,v(nonzeros(Ei)),m,n)' unless options.istrans = 0.  
 
  This function handles the case when v(k) == 0.  For v(k) = 0,
  A(i(k),j(k)) = 0, but As(i(k),j(k)) = 1, and the vector v(eil) provides
  an appropriate input to the edge_weight parameter for all the algorithms.
   
  See the examples reweighted_edges for more information.
 
  ... = indexed_sparse(A,...) takes a set of
  key-value pairs or an options structure.  See set_matlab_bgl_options
  for the standard options. 
     options.undirected: output edge indices for an undirected graph [{0} | 1]
       See the note about undirected inputs.
 
  Note (Undirected inputs): If options.undirected = 1, the input to the
  graph still must contain both undirected edges and the corresponding
  weight.  
 
  Example:
    % see example/reweighted_edges
```

From the documentation, we find that indexed\_sparse has an option called "undirected" which is set to 0 by default.

```
[As A eil Ei] = indexed_sparse(E(:,1), E(:,2), w, n, n, struct('undirected',1));

fprintf('Ei = \n\n');
disp(full(Ei))
```

```
Ei = 

     0     1     0     0     0     0     0     8
     1     0     2     0     0     0     0     0
     0     2     0     3     0     0     0     0
     0     0     3     0     4     0     0     0
     0     0     0     4     0     5     0     0
     0     0     0     0     5     0     6     0
     0     0     0     0     0     6     0     7
     8     0     0     0     0     0     7     0
```

In this case, the indexed\_sparse routine only issued edge indices that were between 1 and 8, rather than 1 and 16 as in the
previous case.

```
[d pred] = shortest_paths(As,u,struct('edge_weight', w(eil)));

path_from_pred(pred,v)
```

```
ans =

     1     8     7     6     5     4     3     2
```

---

## Summary

The functions that support reweighted edges as of MatlabBGL 3.0 are shortest\_paths, all\_shortest\_paths, dijkstra\_sp, bellman\_ford\_sp,
dag\_sp, betweenness\_centrality, astar\_search, johnson\_all\_sp, floyd\_warshall\_all\_sp, mst, kruskal\_mst, and prim\_mst. Note
that max\_flow does not support these indices.

The functions that assist working with the edge indices for the edge\_weight vector are edge\_weight\_index, indexed\_sparse,
and edge\_weight\_vector.

---
