# Supplementary material for: Inferring the effective TOR-dependent network: a computational study in yeast
Source: BMC Syst Biol. 2013 Aug 30;7:84. doi: 10.1186/1752-0509-7-84 (PMC4016608; doi:10.1186/1752-0509-7-84)
Supplement: Additional file 12 — Code/dataset bundle. Compressed ZIP file (*.zip) containing all codes and datasets used in this experiment. [file 1752-0509-7-84-S12.zip › experiment/methods/matlab_bgl/doc/html/core_numbers_example/core_numbers_example.html]

Core numbers in MatlabBGL


 


# Core numbers in MatlabBGL

This example illustrates the concept of the core number of a vertex. Mathematically, the core number of a vertex v is the
largest integer c such that v has degree > 0 when all vertices of degree < c are removed. Equivalently, the core number of
vertex v is the largest integer c such that v exists in a graph where all degrees are at least c.

## Contents

- A simple algorithm
- With MatlabBGL
- The core numbers of a road network

## A simple algorithm

The beauty of core-numbers is that they arey very intuitive to compute and understand.

Let's load some data. This graph comes from the paper with the O(m) algorithm to compute the core numbers of an undirected
graph by Batagelj and Zaversnik, "An O(m) algorithm for the cores decomposition of a network."

```
load '../graphs/cores_example.mat';
```

Plot the data.

```
gplot(A,xy); hold on; plot(xy(:,1), xy(:,2),'r.','MarkerSize',24); hold off;
text(xy(:,1)+0.1, xy(:,2)+0.1, num2str((1:21)'));set(gcf,'Color',[1,1,1]);
set(gca,'XTick',[]);set(gca,'YTick',[]);xlim([-1,10]);ylim([-2,7]);axis off;
```

By inspection, vertex 16 is in a 0 core because it has no edges in the graph.

From the statement of the property, let's figure out the core numbers for this graph. For each possible degree d, let's remove
all vertices with degree <= d and degree>0 and repeat this until there are no vertices with degree (0,d]. Then, any vertex
that is left, must have core number at least d+1.

The following code implements that algorithm where the graph Ad is the current working version of the graph. At the end of
the for loop, the graph Ad is the graph A where all vertices of degree <= d have been removed.

```
max_deg = full(max(sum(A)));
cn = zeros(num_vertices(A),1);
Ad = A; dv=sum(Ad);
for d=0:max_deg
  % while they are vertices with degree in (0,d], remove them
  while any(dv<=d&dv>0), Ad(dv<=d,:)=0;Ad(:,dv<=d)=0;dv=sum(Ad);end
  % any vertex that is left must core number at least d+1;
  cn(dv>d) = d+1;
end
arrayfun(@(v,c) fprintf('core_number(%2i) = %i\n',v,c),1:size(A,1),cn');
```

```
core_number( 1) = 1
core_number( 2) = 2
core_number( 3) = 2
core_number( 4) = 3
core_number( 5) = 3
core_number( 6) = 3
core_number( 7) = 3
core_number( 8) = 3
core_number( 9) = 2
core_number(10) = 3
core_number(11) = 2
core_number(12) = 1
core_number(13) = 1
core_number(14) = 3
core_number(15) = 3
core_number(16) = 0
core_number(17) = 2
core_number(18) = 2
core_number(19) = 2
core_number(20) = 2
core_number(21) = 1
```

The output shows us that vertex 16 is the only vertex with a core number of 0. Because this graph is an example, "it just
so happens" that the cores make a nice picture. The following code plots the convex hull around the points inside of a k-core.
The darker the color, the higher the core.

```
clf; hold on; wh=ones(1,3); colors={0.85*wh,0.7*wh,0.55*wh,0.4*wh};
for c=unique(cn')
  m=cn>=c; cl=colors{c+1}; lw=16*1.5^(max(cn)-c);
  xym=xy(m,:);is=convhull(xym(:,1),xym(:,2));
  h=fill(xym(is,1),xym(is,2),cl);set(h,'LineWidth',lw,'EdgeColor',cl);
end
gplot(A,xy,'k-');  plot(xy(:,1), xy(:,2),'r.','MarkerSize',24);
text(xy(:,1)+0.1, xy(:,2)+0.1, num2str((1:21)'));set(gcf,'Color',[1,1,1]);
xlim([-1,10]);ylim([-2,7]);axis off; hold off;
```

The figure shows the 0-core, the 1-core, the 2-core and the 3-core. Although vertex 11 has degree 5, it is only a 2-core vertex
because it links to two 1-core vertices. Also, note that the cores aren't necessarily connected components. The 2-core contains
two connected components (2-11,14-15) and (17-20).

---

## With MatlabBGL

The MatlabBGL function core\_numbers implements efficient algorithms to compute the cores of a graph. These algorithms are
significantly more efficient than the previous code and produce identical output.

Let's check that they produce the same output.

```
cn_bgl = core_numbers(A);
any(cn_bgl-cn)
```

```
ans =

     0
```

---

## The core numbers of a road network

For fun, let's see how many cores there are in a road network. Vertices in a 1-core in a road network have at least one path
between them (assuming the underlying network is connected). Vertices in a 2-core have at least two paths between them.

```
load('../graphs/minnesota.mat');A=spones(A); % convert to unweighted
[cn csz]=core_numbers(A);  cs=unique(cn);
arrayfun(@(v,c) fprintf('core(%2i) = %4i\n',v,c),cs,csz(cs+1));
% Now, you might complain that there are certain vertices in this graph
% that simply chain a path in the road network so it draws correctly.  The
% following code removes all vertices of degree two and connects the
% end-points of the degree two vertex directly.  It applies this prodecure
% iteratively until all the vertices of degree two are gone.  At the end,
% we compute the core nubmers again.
d2v=find(sum(A,2)==2);
while ~isempty(d2v)
    for v=d2v'
        l=find(A(:,v));A(l(1),v)=0;A(l(2),v)=0;A(v,l(1))=0;A(v,l(2))=0;
        A(l(1),l(2))=1;A(l(2),l(1))=1;
    end
    d2v=find(sum(A,2)==2);
end
max(core_numbers(A))
```

```
core( 1) =    2
core( 2) =   98

ans =

     2
```

The highest core is still two! I think that's pretty amazing. There aren't subnetworks of the Minnesota highways where you
always have at least three choices at every intersection.

---
