# Supplementary material for: Inferring the effective TOR-dependent network: a computational study in yeast
Source: BMC Syst Biol. 2013 Aug 30;7:84. doi: 10.1186/1752-0509-7-84 (PMC4016608; doi:10.1186/1752-0509-7-84)
Supplement: Additional file 12 — Code/dataset bundle. Compressed ZIP file (*.zip) containing all codes and datasets used in this experiment. [file 1752-0509-7-84-S12.zip › experiment/methods/matlab_bgl/doc/html/old.html]

MatlabBGL - Versions


# MatlabBGL

## A Matlab Graph Library

### MatlabBGL

- Main
- FAQ
- Examples
- Change Log
- Documentation
- Older Versions
- Launchpad page
- Download

## Older Versions

MatlabBGL 3.0-beta. (Documentation not available for beta version.)

MatlabBGL 2.0 and
Matlab BGL 2.0 documentation

MatlabBGL 1.01 and
Matlab BGL 1.0 documentation

MatlabBGL 1.0 and
Matlab BGL 1.0 documentation

FAQ | Documentation | Older Versions

... back to website.

Copyright 2006-2007, David Gleich
