# Supplementary material for: Inferring the effective TOR-dependent network: a computational study in yeast
Source: BMC Syst Biol. 2013 Aug 30;7:84. doi: 10.1186/1752-0509-7-84 (PMC4016608; doi:10.1186/1752-0509-7-84)
Supplement: Additional file 12 — Code/dataset bundle. Compressed ZIP file (*.zip) containing all codes and datasets used in this experiment. [file 1752-0509-7-84-S12.zip › experiment/methods/matlab_bgl/doc/html/index.html]

MatlabBGL


# MatlabBGL

## A Matlab Graph Library

### MatlabBGL

- Main
- FAQ
- Examples
- Change Log
- Documentation
- Older Versions
- Launchpad page
- Download

MatlabBGL is a Matlab package for working with graphs.  It uses the 
Boost Graph Library to efficiently implement the graph algorithms. 
MatlabBGL
is designed to work with large sparse graphs with hundreds of thousands of nodes.

Written by David Gleich.

---

## News

2008-10-21: Version 4.0 finished. Major updates.
  
 MatlabBGL Changelog.
Download MatlabBGL 4.0!

2007-07-22: Version 3.0-beta released. Major updates. 
  
MatlabBGL Changelog. Download MatlabBGL 3.0!
(The final version will be released on the Mathworks File Exchange.)

2007-04-11: Version 2.1 released. Minor updates. 
  
MatlabBGL Changelog.

2006-07-25: Version 2.0 released. Significant updates. 
  
MatlabBGL Changelog.

2006-05-09: Version 1.01 released. Fixes minor bugs. 
  
MatlabBGL Changelog.

## Getting MatlabBGL (Latest Version: 4.0)

The files for MatlabBGL reside on the Mathworks Matlab Central File Exchange. 
The lastest version is always there.

Go to the 
MatlabBGL page at the Matlab File Exchange.

To install MatlabBGL,

1. Download the latest link from the File Exchange and unzip it to a directory of your
   choosing.
2. Open Matlab and change directory until you get to the directory where you unzipped
   it.
3. Change into the matlab\_bgl subdirectory.
4. Try typing 
   clustering\_coefficients(sparse(ones(5))) into Matlab.  You should see
   the following output.  

   ```
   ans =

        1
        1
        1
        1
        1
   ```

If the above steps do not work, please send me an email with any error messages
you get and your system configuration.

## Documentation

The documentation file is out of date. I apologize. The help
commands are up to date. Also, the examples are up to date.

Shortcut: MatlabBGL 2.1 Documentation

In the future, we hope to have online documentation.  Right now, the documentation
associated with MatlabBGL is a pdf file distributed with the library and linked
above.

## Limitations

Right now, the library only works with Matlab 7.0 and above.

### Testing matrix

I have tested MatlabBGL 4.0 with every system in green.
Entries in blue have been tested but require recompiling libmbgl.

|  | Win32 | Win64 | Linux32 | Linux64 | MacPPC | Maci386 |
| --- | --- | --- | --- | --- | --- | --- |
| Matlab 7.0 |  |  |  |  |  |  |
| Matlab 7.1 SP3 |  |  |  |  |  |  |
| Matlab R2006a |  |  |  |  |  |  |
| Matlab R2006b |  |  |  |  |  |  |
| Matlab R2007a |  |  |  |  |  |  |
| Matlab R2007b |  |  |  |  |  |  |
| Matlab R2008a |  |  |  |  |  |  |
| Matlab R2008b |  |  |  |  |  |  |

## Have Questions?

See the MatlabBGL FAQ, it may have answers!

Alternatively, you should contribute a
matlab-bgl question on Launchpad.

## Examples

- Finding the red-black ordering of a
  matrix.
- Recording the behavior of an algorithm.
- An explanation of the cores of a graph.
- Using edge reweighting to run algorithms with 0 edge weights.
- Working with planar graphs.

- New features in MatlabBGL-3.
- New features in MatlabBGL-4.

## Problems

Do you have a problem with MatlabBGL?  Are you getting an error?  Please
send me a .mat file with your example graph and a list of commands to reproduce
the error.

Send both of these to the address mithandor+mbglerrors@gmail.com.

## Contributing

Checkout the 
matlab-bgl Launchpad page to get all the code!

## Acknowledgements

Thanks to the Boost Graph Library team for a useful and helpful product.  Also,
the ICME Students who helped me test the software and Amin Saberi for giving me
a little while to work on it. Many
thanks to all the folks who sent in bugs with the library.

FAQ | Documentation | Older Versions

... back to website.

Copyright 2006-2007, David Gleich
