# Supplementary material for: Inferring the effective TOR-dependent network: a computational study in yeast
Source: BMC Syst Biol. 2013 Aug 30;7:84. doi: 10.1186/1752-0509-7-84 (PMC4016608; doi:10.1186/1752-0509-7-84)
Supplement: Additional file 12 — Code/dataset bundle. Compressed ZIP file (*.zip) containing all codes and datasets used in this experiment. [file 1752-0509-7-84-S12.zip › experiment/methods/matlab_bgl/doc/html/faq.html]

MatlabBGL - FAQ


# MatlabBGL

## A Matlab Graph Library

### MatlabBGL

- Main
- FAQ
- Examples
- Change Log
- Documentation
- Older Versions
- Launchpad page
- Download

## FAQ

---

- Help...? (Something doesn't work.)
- Does it work...? (Platform Questions)
- How do I...? (Usage Questions)
- What does...? (Extra Help)
- But what bugs...? (Known Bugs)

---


#### Help...?

1. ... Matlab just crashes when I try calling some of your routines!

   In general, this happens on Linux.  One problem I've found is that the version
   of gcc I compiled Matlab with is not always the best one.  If you reliably
   have this problem, I can look into using a new compiler, so please, let me know. 
   If you need a short term fix, I believe this page addresses the issue.

Mathworks Help page on mexErrMsgTxt

2. ... Matlab gives me an error about 64-bit mex files and sparse matrix compatibility.

   The error I get from Matlab is

   ```
   ??? Function "mxGetIr_700" is obsolete in file "compat32.cpp", line 246.
   (64-bit mex files using sparse matrices must be rebuilt with the "-largeArrayDims" option. ...)
   ```

   The solution to this error is to update to Matlab BGL 2.1 which includes
   precompiled files for 64-bit linux with support for largeArrayDims included.
3. ... I get an error about "undefined symbol: mxCreateDoubleMatrix\_700."

   This error results because I have compiled MatlabBGL for a more recent version of Matlab than
   the one you are using. The fix is straightforward: you need to recompile the library for
   your version of Matlab.

   ```
   >> cd matlab_bgl % or switch to the directory with all the matlab_bgl files

   >> cd private
   >> compile
   ```

   If this solution does not work, please send me email with your error message as well
   as the output of the ver command from Matlab.
4. ... MatlabBGL crashes when I call "max\_flow."

   This error has been fixed as of MatlabBGL 3.0. This fix applies to earlier versions. With some combinations of Matlab and libc, there are bad interactions with the
   free function used inside of a mex file. The "private/max\_flow\_mex.c" file inadvertantly
   used this function to release memory allocated by Matlab. Again, the fix is
   simple: replace the "free" call with "mxFree".

   ```
   >> cd matlab_bgl % or switch to the directory with all the matlab_bgl files

   >> cd private
   >> edit max_flow_mex.c
   (in editor) ... Edit->Find and Replace...
   (in find/replace) ... Find what: free; Replace with: mxFree; Match case (on)
   (in find/replace) Replace All
   (in editor) ... File->Save
   >> compile
   ```

   If this solution does not work, please send me email with your error message as well
   as the output of the ver command from Matlab.

#### Does it work...?

1. ... with Linux?

   It should!  I've tested it on a few versions and it has worked assuming you
   have Matlab 7.0 or higher. The code works on both 32-bit and 64-bit version
   of Linux. The precompiled files for 64-bit Linux require Matlab 2006b
   because of a change in the sparse matrix data structure, however, you
   can recompile the files yourself for previous versions of Matlab.
2. ... with Windows?

   Yes. I develop the software on Windows. You don't need to do anything (I hope!) to make
   it work on 32-bit versions of Windows and Matlab. For 64-bit versions, you will have to
   compile some things yourself because I don't have a 64-bit computer with Windows and
   Matlab installed. Please email me if you have questions about a 64-bit version of
   Windows.
3. ... with MacOS?

   Yes, as of version 2.1, it works! For the Intel processor, you will have to compile
   some things yourself because I don't have access to an Intel Mac with Matlab.
4. ... with Sun?

   I have gotten this to work, you should be able to adapt the compile scripts to
   work for sun..
5. ... on 64-bit Matlab?

   Yes!

---


#### How do I...?

1. ... create a graph?

   Any Matlab sparse matrix is a MatlabBGL graph.  See the Matlab document on
   sparse matrices.  The sparse matrix IS the adjacency matrix of the graph.
2. ... add graph properties?

   MatlabBGL doesn't support graph propreties like Boost does.  Instead, it allows
   you to store separate arrays with data about each vertex.  This technique is
   how all the labels and coordinates are done in the examples.  Eventually, I'll
   put up a tutorial with more examples of how to use this idea.  (Send me email
   if you need it...)
3. ... make the matrix/graph symmetric?

   If you have an undirected graph, then MatlabBGL needs both the forward and reverse
   edge listed.  This requirement means that the adjacency matrix must be symmetric. 
   The easiest way to make a sparse Matlab matrix symmetric is to call

   ```
   A = max(A,A');
   ```
4. ... make an unweighted graph?

   If you have a weighted graph and want to make an unweighted graph, there are two
   options.  First, for MatlabBGL calls that do not use graph weights, you do
   not need to do anything!  These algorithms (e.g. bfs)
   will not look at the weights on your graph so it will behave exactly like an unweighted
   graph.  If the algorithm does look at the weights (e.g. dijkstra\_sp)
   then you can create an unweighted version with the following call

   ```
   A = spones(A);
   ```
5. ... use a dense graph

   Right now, you can't.  All the MatlabBGL algorithms only work on sparse Matlab
   matrices.  You can convert your dense graph into a sparse matrix using the
   command

   ```
   A = sparse(A);
   ```

---


#### What does...?

1. ... "the matrix A must..." mean?

   MatlabBGL errors are often in terms of "the matrix A."  The matrix A is the
   adjacency matrix for the graph.  These errors mean that there is some problem
   with your input matrix.
2. ... "the matrix A must be sparse" mean?

   MatlabBGL only uses sparse matrices by default.  You can set an option to convert
   all full matrices to sparse matrices.

   ```
   set_matlab_bgl_default('full2sparse',1);
   ```

   If you want to permanently set full2sparse then
   edit set\_matlab\_bgl\_default.m and change

   ```
   default_options = struct('istrans', 0, 'nocheck', 0, 'full2sparse', 0);
   ```

   to

   ```
   default_options = struct('istrans', 0, 'nocheck', 0, 'full2sparse', 1);
   ```
3. ... "the matrix A must have double values" mean?

   For algorithms that use the weights on the graph edges, those weights must be double
   values.  Sometimes, you can inadvertently create a Matlab sparse matrix with
   a different value type.  To convert your graph to a double type, use the statement

   ```
   A = double(A);
   ```
4. ... "the matrix A must be symmetric" mean?

   Certain algorithms require undirected graphs.  For these algorithms, 
   your adjacency matrix must be symmetric.  See How do I make my matrix symmetric
   in the "How do I...?" section.
5. ... "the matrix A must be square" mean?

   The input to a MatlabBGL function is an adjacency matrix.  By definition, an
   adjacency matrix is square.  This error probably means you are passing something
   to a MatlabBGL call incorrectly.

---


#### What what bugs does the software have?

1. Presently, there are some problems with various calls on empty matrices with 64-bit matlab.
   For example

   ```
   T = mst(sparse([]));
   ```

   will fail with an out of memory error.
   These bugs will be fixed in the next version. If you need a fix before then, contact me for
   the lastest release. You will likely need to recompile files for your system.

FAQ | Documentation | Older Versions

... back to website.

Copyright 2006-2007, David Gleich
