# Supplementary material for: Inferring the effective TOR-dependent network: a computational study in yeast
Source: BMC Syst Biol. 2013 Aug 30;7:84. doi: 10.1186/1752-0509-7-84 (PMC4016608; doi:10.1186/1752-0509-7-84)
Supplement: Additional file 12 — Code/dataset bundle. Compressed ZIP file (*.zip) containing all codes and datasets used in this experiment. [file 1752-0509-7-84-S12.zip › experiment/methods/matlab_bgl/doc/html/changes.html]

MatlabBGL - Changes


# MatlabBGL

## A Matlab Graph Library

### MatlabBGL

- Main
- FAQ
- Examples
- Change Log
- Documentation
- Older Versions
- Launchpad page
- Download

## Changes

Version 4.0

- Added graph layout algorithms.
- Added planar graph algorithms.
- Improved testing code.
- Upgraded to boost 1.36.0.
- Added edge\_weight\_vector function.
- New option syntax.
- Fixed bugs with graph generation routines.

Version 3.1 (Internal release)

- Fixed bug with predecessor return from floyd\_warshall\_all\_sp.
- Updated documentation for all\_shortest\_paths for predecessor return.

Version 3.0

- Added grid\_graph function.
- Added core\_numbers function.
- Added weighted and directed clustering coefficients function.
- Added lengauer\_tarjan\_dominator\_tree function.
- Added core\_numbers\_example.
- Added matching, edmonds\_maximum\_cardinality\_matching, maximal\_matching, and test\_matching functions.
- Added kolmogorov\_max\_flow and edmunds\_karp\_max\_flow functions.
- Added topological\_order function.
- Added pred\_from\_path to convert predecessors into a path.
- Added edge\_weight\_index function to automatically generate edge\_weight indices.
- Added 'target' vertex option to all shortest path and search algorithms to stop the search
  early if it finds a target vertex.
- Added edge\_weight option to all functions that depend on a weighted graph except max\_flow.
  This option allows edge weights to be separate from the graph and allows 0 edge weights.
- Added signifcantly more testing code.
- Added new examples for edge-reweighted graphs.
- Updated to Boost 1.34.0.
- Removed kludge to fix bug in Johnson's all pairs shortest path from Boost 1.33.1.
- max\_flow, floyd\_warshall, and betweenness\_centrality now correctly implement the transpose option.
- Fixed bug with breadth\_first\_search not stopping.
- Fixed documentation bug with breadth\_first\_search function.
- Fixed compiler warnings without -ansi on g++-4.0
- Fixed bugs with out of range vertices in all shortest path algorithms.

Version 2.1

- Fixed bug with MST and disconnected graphs.
- Added edge centrality output to betweeneness\_centrality.
- Added predecessor matrix to floyd\_warshall all pairs shortest paths
- Updated sparse matrix interface to work with Matlab 2006b on 64-bit processors
- Fixed error with erdos\_reyni
- Added graph generation functions star\_graph, cycle\_graph, wheel\_graph

Version 2.0

- Added support for visitors.
- Added astar\_search.
- Added some trivial functions (num\_edges, num\_vertices).
- Added erdos\_reyni graph creation.
- Fixed symmetrization error with MST and negative edge weights.
- Added support for non-sparse input.
- Added additional examples.

Version 1.01

- Fixed error with mst.m and graphs with negative weights.
- Changed default MST algorithm to kruskal.
- Fixed error with components\_mex.c and computing component sizes incorrectly.
- Added debugging code to library.
- Added regression tests.

FAQ | Documentation | Older Versions

... back to website.

Copyright 2006-2007, David Gleich
