# Supplementary figures and images for: Inferring the effective TOR-dependent network: a computational study in yeast
Source: BMC Syst Biol. 2013 Aug 30;7:84. doi: 10.1186/1752-0509-7-84 (PMC4016608; doi:10.1186/1752-0509-7-84)

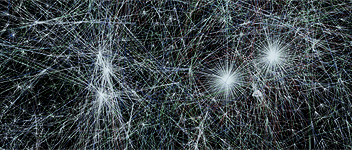

Supplement: Additional file 12 — Code/dataset bundle. Compressed ZIP file (*.zip) containing all codes and datasets used in this experiment. [file 1752-0509-7-84-S12.zip › experiment/methods/matlab_bgl/doc/html/images/matlab-bgl-header.png]

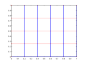

Supplement: Additional file 12 — Code/dataset bundle. Compressed ZIP file (*.zip) containing all codes and datasets used in this experiment. [file 1752-0509-7-84-S12.zip › experiment/methods/matlab_bgl/doc/html/new_in_3/new_in_3_0.png]

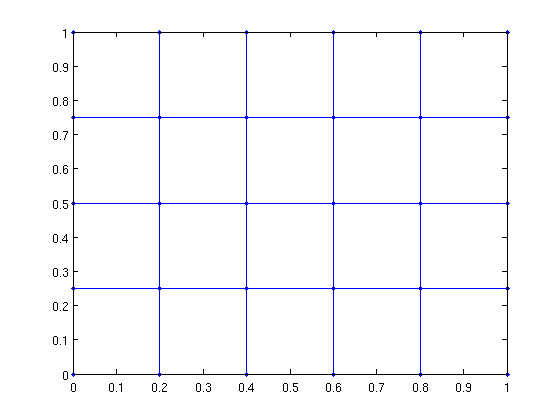

Supplement: Additional file 12 — Code/dataset bundle. Compressed ZIP file (*.zip) containing all codes and datasets used in this experiment. [file 1752-0509-7-84-S12.zip › experiment/methods/matlab_bgl/doc/html/new_in_3/new_in_3_0_01.png]

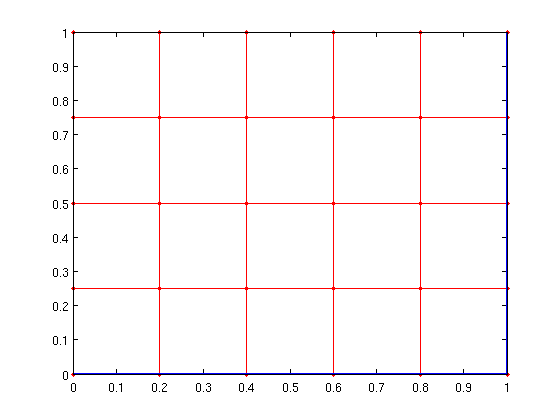

Supplement: Additional file 12 — Code/dataset bundle. Compressed ZIP file (*.zip) containing all codes and datasets used in this experiment. [file 1752-0509-7-84-S12.zip › experiment/methods/matlab_bgl/doc/html/new_in_3/new_in_3_0_02.png]

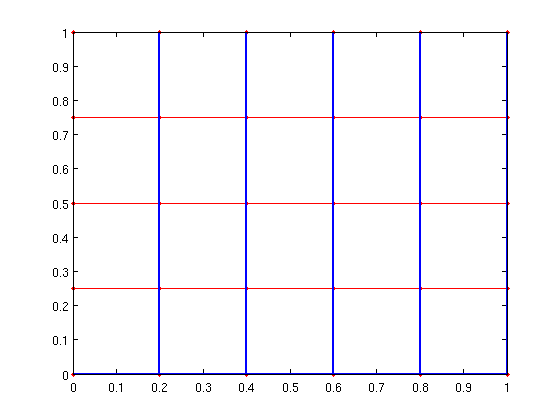

Supplement: Additional file 12 — Code/dataset bundle. Compressed ZIP file (*.zip) containing all codes and datasets used in this experiment. [file 1752-0509-7-84-S12.zip › experiment/methods/matlab_bgl/doc/html/new_in_3/new_in_3_0_03.png]

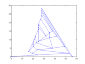

Supplement: Additional file 12 — Code/dataset bundle. Compressed ZIP file (*.zip) containing all codes and datasets used in this experiment. [file 1752-0509-7-84-S12.zip › experiment/methods/matlab_bgl/doc/html/new_in_4/new_in_4_0.png]

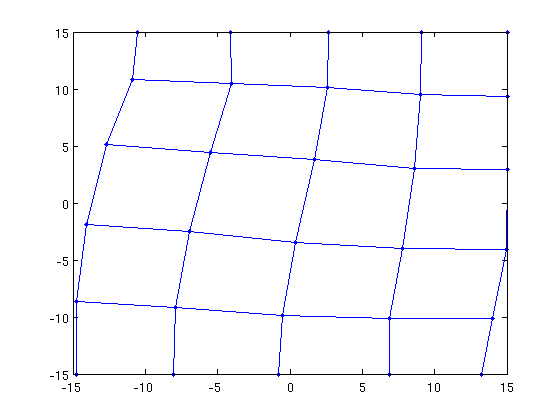

Supplement: Additional file 12 — Code/dataset bundle. Compressed ZIP file (*.zip) containing all codes and datasets used in this experiment. [file 1752-0509-7-84-S12.zip › experiment/methods/matlab_bgl/doc/html/new_in_4/new_in_4_0_02.png]

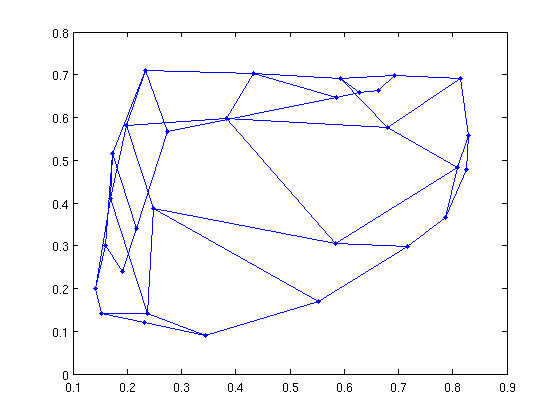

Supplement: Additional file 12 — Code/dataset bundle. Compressed ZIP file (*.zip) containing all codes and datasets used in this experiment. [file 1752-0509-7-84-S12.zip › experiment/methods/matlab_bgl/doc/html/new_in_4/new_in_4_0_03.png]

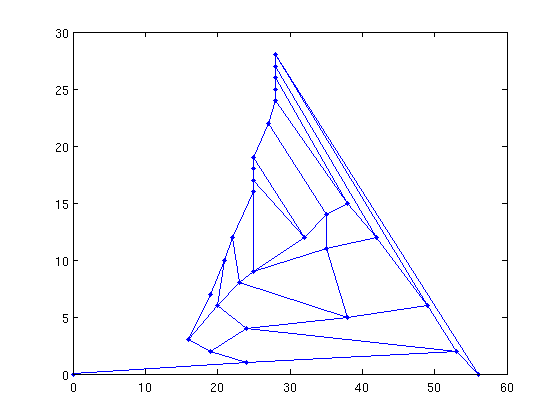

Supplement: Additional file 12 — Code/dataset bundle. Compressed ZIP file (*.zip) containing all codes and datasets used in this experiment. [file 1752-0509-7-84-S12.zip › experiment/methods/matlab_bgl/doc/html/new_in_4/new_in_4_0_04.png]

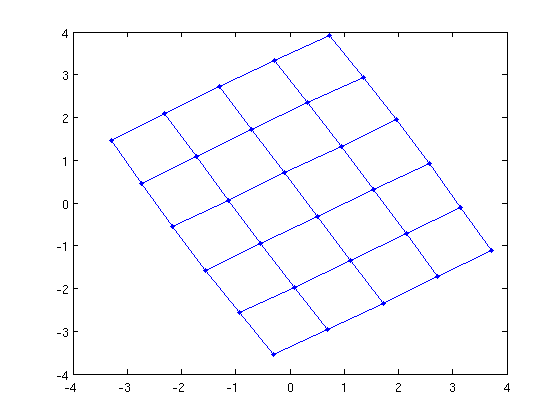

Supplement: Additional file 12 — Code/dataset bundle. Compressed ZIP file (*.zip) containing all codes and datasets used in this experiment. [file 1752-0509-7-84-S12.zip › experiment/methods/matlab_bgl/doc/html/new_in_4/new_in_4_0_01.png]

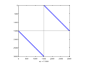

Supplement: Additional file 12 — Code/dataset bundle. Compressed ZIP file (*.zip) containing all codes and datasets used in this experiment. [file 1752-0509-7-84-S12.zip › experiment/methods/matlab_bgl/doc/html/red_black/red_black.png]

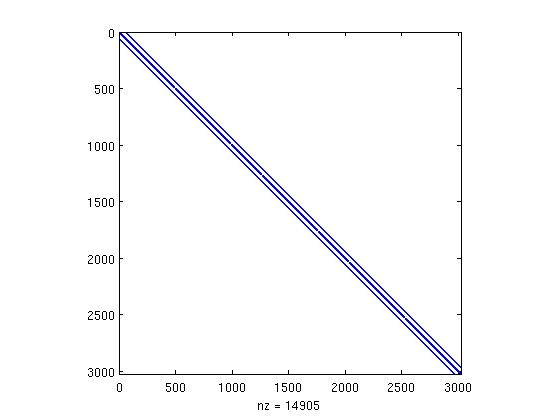

Supplement: Additional file 12 — Code/dataset bundle. Compressed ZIP file (*.zip) containing all codes and datasets used in this experiment. [file 1752-0509-7-84-S12.zip › experiment/methods/matlab_bgl/doc/html/red_black/red_black_01.png]

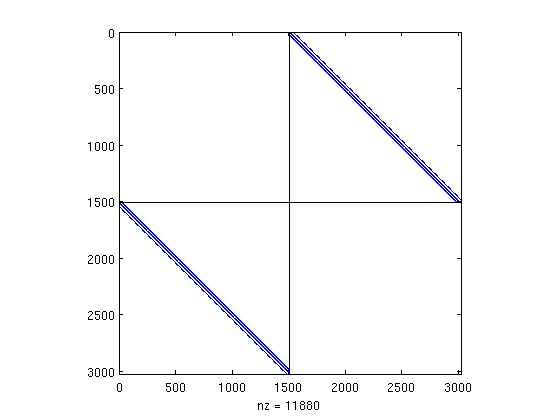

Supplement: Additional file 12 — Code/dataset bundle. Compressed ZIP file (*.zip) containing all codes and datasets used in this experiment. [file 1752-0509-7-84-S12.zip › experiment/methods/matlab_bgl/doc/html/red_black/red_black_04.png]

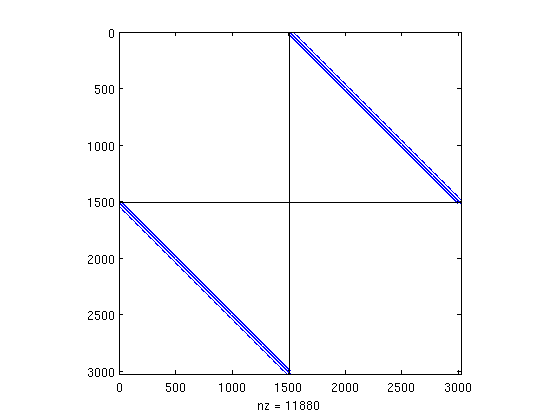

Supplement: Additional file 12 — Code/dataset bundle. Compressed ZIP file (*.zip) containing all codes and datasets used in this experiment. [file 1752-0509-7-84-S12.zip › experiment/methods/matlab_bgl/doc/html/red_black/red_black_03.png]

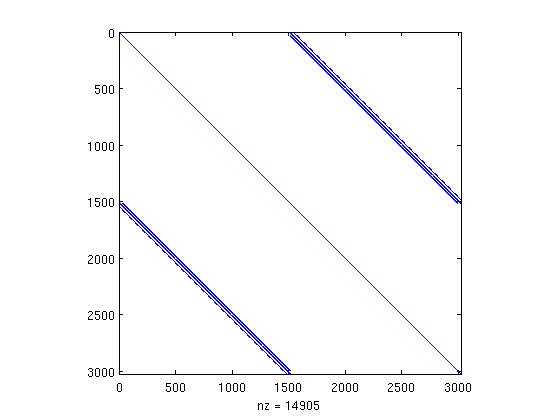

Supplement: Additional file 12 — Code/dataset bundle. Compressed ZIP file (*.zip) containing all codes and datasets used in this experiment. [file 1752-0509-7-84-S12.zip › experiment/methods/matlab_bgl/doc/html/red_black/red_black_02.png]

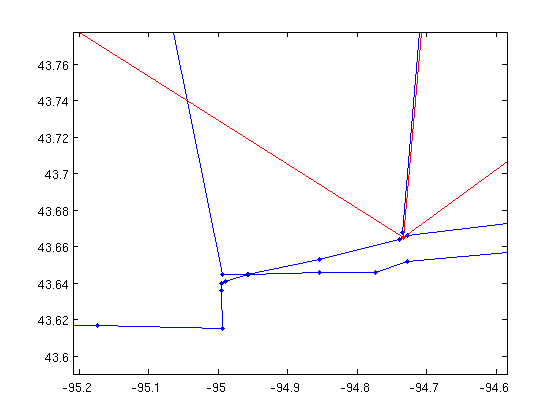

Supplement: Additional file 12 — Code/dataset bundle. Compressed ZIP file (*.zip) containing all codes and datasets used in this experiment. [file 1752-0509-7-84-S12.zip › experiment/methods/matlab_bgl/doc/html/planar_graphs/planar_graphs_05.png]

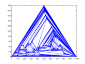

Supplement: Additional file 12 — Code/dataset bundle. Compressed ZIP file (*.zip) containing all codes and datasets used in this experiment. [file 1752-0509-7-84-S12.zip › experiment/methods/matlab_bgl/doc/html/planar_graphs/planar_graphs.png]

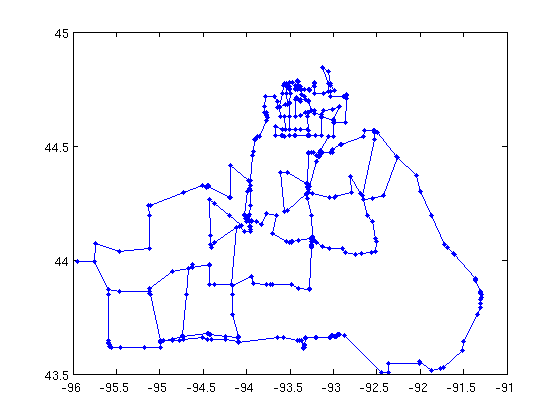

Supplement: Additional file 12 — Code/dataset bundle. Compressed ZIP file (*.zip) containing all codes and datasets used in this experiment. [file 1752-0509-7-84-S12.zip › experiment/methods/matlab_bgl/doc/html/planar_graphs/planar_graphs_03.png]

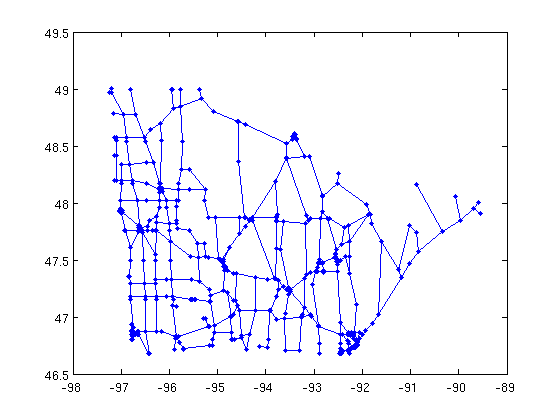

Supplement: Additional file 12 — Code/dataset bundle. Compressed ZIP file (*.zip) containing all codes and datasets used in this experiment. [file 1752-0509-7-84-S12.zip › experiment/methods/matlab_bgl/doc/html/planar_graphs/planar_graphs_06.png]

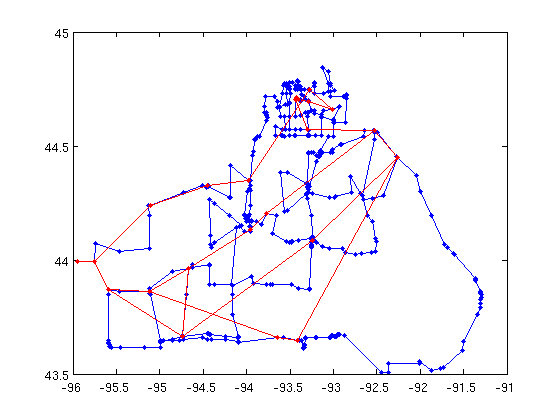

Supplement: Additional file 12 — Code/dataset bundle. Compressed ZIP file (*.zip) containing all codes and datasets used in this experiment. [file 1752-0509-7-84-S12.zip › experiment/methods/matlab_bgl/doc/html/planar_graphs/planar_graphs_04.png]

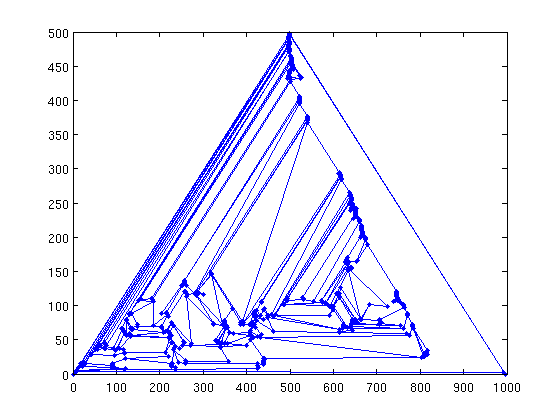

Supplement: Additional file 12 — Code/dataset bundle. Compressed ZIP file (*.zip) containing all codes and datasets used in this experiment. [file 1752-0509-7-84-S12.zip › experiment/methods/matlab_bgl/doc/html/planar_graphs/planar_graphs_07.png]

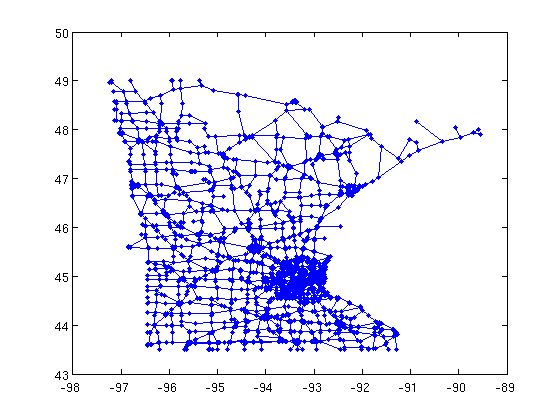

Supplement: Additional file 12 — Code/dataset bundle. Compressed ZIP file (*.zip) containing all codes and datasets used in this experiment. [file 1752-0509-7-84-S12.zip › experiment/methods/matlab_bgl/doc/html/planar_graphs/planar_graphs_01.png]

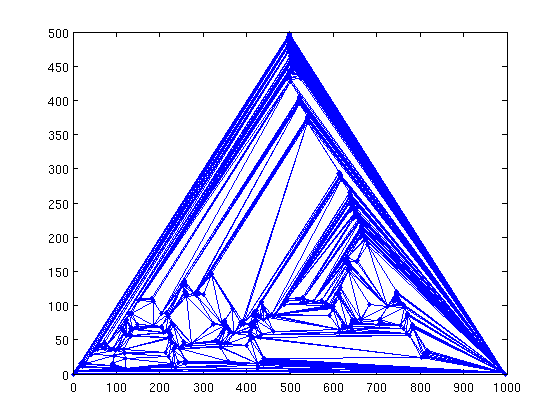

Supplement: Additional file 12 — Code/dataset bundle. Compressed ZIP file (*.zip) containing all codes and datasets used in this experiment. [file 1752-0509-7-84-S12.zip › experiment/methods/matlab_bgl/doc/html/planar_graphs/planar_graphs_08.png]

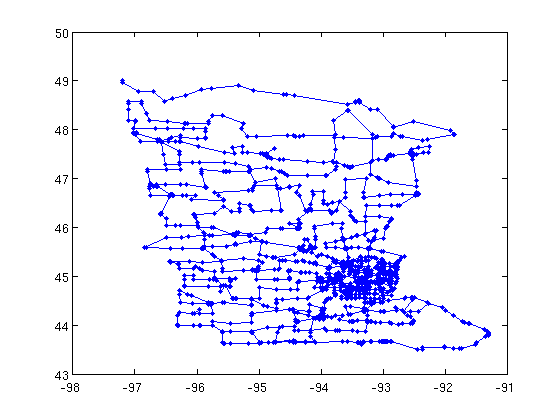

Supplement: Additional file 12 — Code/dataset bundle. Compressed ZIP file (*.zip) containing all codes and datasets used in this experiment. [file 1752-0509-7-84-S12.zip › experiment/methods/matlab_bgl/doc/html/planar_graphs/planar_graphs_02.png]

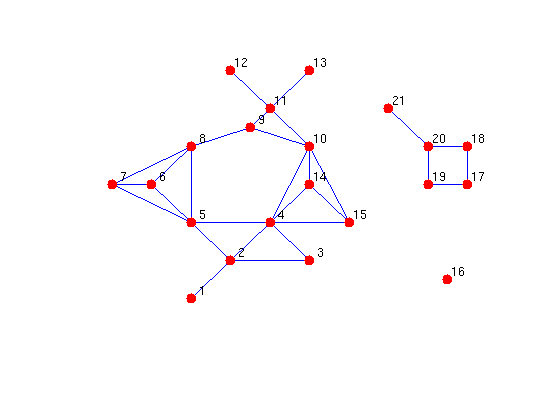

Supplement: Additional file 12 — Code/dataset bundle. Compressed ZIP file (*.zip) containing all codes and datasets used in this experiment. [file 1752-0509-7-84-S12.zip › experiment/methods/matlab_bgl/doc/html/core_numbers_example/core_numbers_example_01.png]

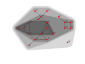

Supplement: Additional file 12 — Code/dataset bundle. Compressed ZIP file (*.zip) containing all codes and datasets used in this experiment. [file 1752-0509-7-84-S12.zip › experiment/methods/matlab_bgl/doc/html/core_numbers_example/core_numbers_example.png]

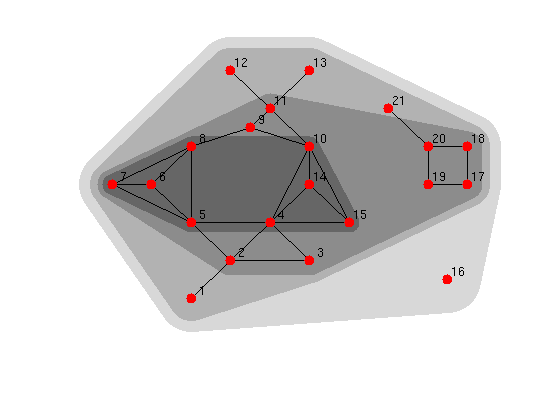

Supplement: Additional file 12 — Code/dataset bundle. Compressed ZIP file (*.zip) containing all codes and datasets used in this experiment. [file 1752-0509-7-84-S12.zip › experiment/methods/matlab_bgl/doc/html/core_numbers_example/core_numbers_example_02.png]
